# Supplementary material for: Identification and improvement of isothiocyanate-based inhibitors on stomatal opening to act as drought tolerance-conferring agrochemicals
Source: Nat Commun. 2023 May 15;14:2665. doi: 10.1038/s41467-023-38102-7 (PMC10185662; doi:10.1038/s41467-023-38102-7)
Supplement: Supplementary file 1 — Supplementary Information File [file 41467_2023_38102_MOESM1_ESM.pdf]

Supplementary Information for

Identification and improvement of isothiocyanate-based inhibitors on stomatal opening to act as drought tolerance–conferring agrochemicals

Yusuke Aihara, Bumpei Maeda, Kanna Goto, Koji Takahashi, Mika Nomoto, Shigeo Toh, Wenxiu Ye, Yosuke Toda, Mami Uchida, Eri Asai, Yasuomi Tada, Kenichiro Itami, Ayato Sato, Kei Murakami* and Toshinori Kinoshita*

*Toshinori Kinoshita Email**:** kinoshita@bio.nagoya-u.ac.jp

*Kei Murakami Email: kei.murakami@kwansei.ac.jp

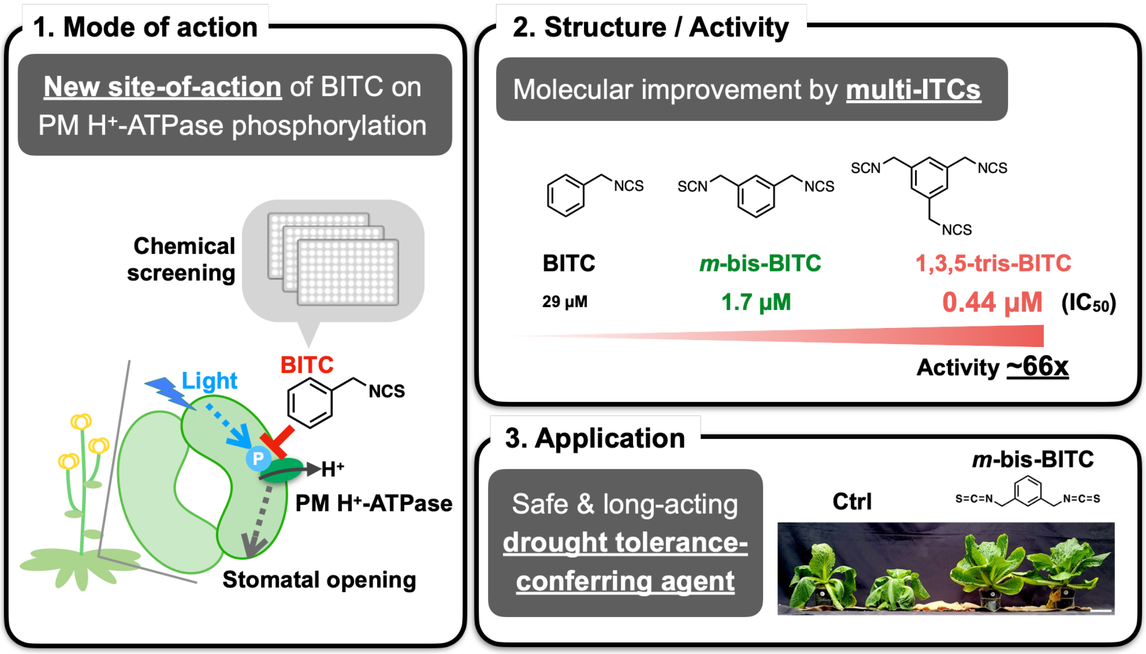

Supplementary Fig. 1. Graphical summary of the findings of this study.

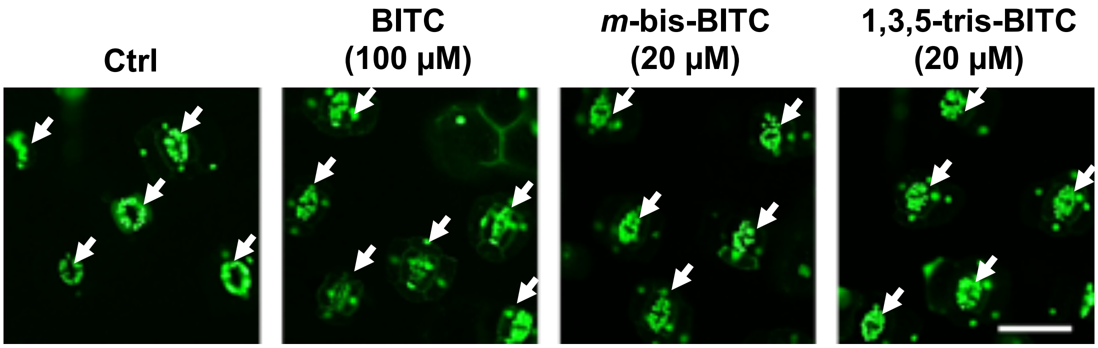

Supplementary Fig. 2. The viability of *C. benghalensis* guard cells after BITC treatment.
C. benghalensis leaf discs were treated for 3–4 h with BITC or its derivatives at the indicated concentrations. Afterward, the abaxial epidermis was peeled and treated with 1 μg/mL FDA. The fluorescence of the guard cells (arrows) is caused by their esterase activity, which hydrolyzes FDA to produce fluorescein. The images shown are representative of an experiment that was replicated twice with different biological samples. Bar = 100 μm.

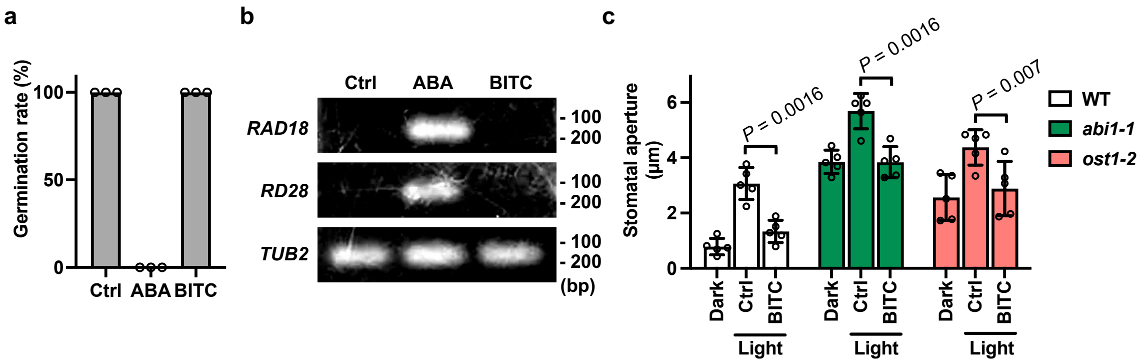

Supplementary Fig. 3. Effect of BITC on the ABA-related physiological response in *A. thaliana*.

(**a**) The effects of BITC on *Arabidopsis* seed germination. Seeds were treated with water containing 50 μM ABA or BITC or an equal volume of DMSO (Ctrl) and incubated at 22˚C under a photoperiod of 16-h white light (50 μmol m^{-2} s^{-1})/8-h dark. Seed germination ratios were calculated 7 days after treatment. Values are presented as mean ± SD (n =3; 30 seeds per replicate). (**b**) The effect of BITC on ABA-responsive gene expression in *Arabidopsis* seedlings, *RAB18* and RD29B. For 3 h, the seedlings were treated with 50 μM ABA or BITC or an equal volume of DMSO (Ctrl) at 24˚C. Representative data set from an experiment replicated three times with different biological samples are shown. (**c**) Effect of BITC (50 μM) on light-induced stomatal opening in Arabidopsis mutants insensitive to ABA. Values are presented as means ± SD (n = 5; 46–109 stomata from three leaf discs per replicate). *P*-values are indicated (paired, two-sided Student's t-tests).

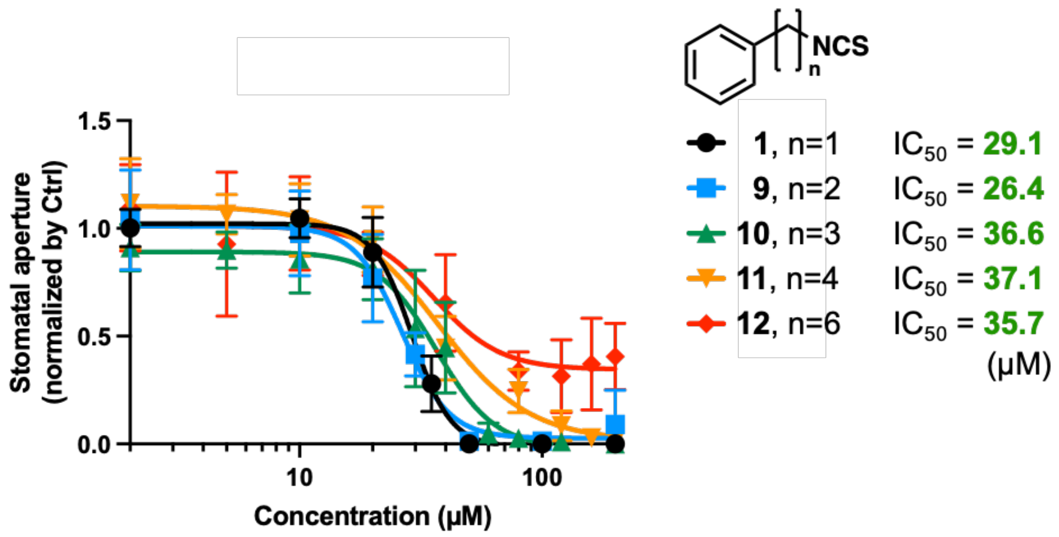

Supplementary Fig. 4. Dose-dependent effect of BITC derivatives with varying alkyl linker lengths (1, 9–12).

The BITC derivatives were tested for their ability to inhibit stomatal opening in *C. benghalensis*, as shown in Figure 1B. Values are in comparison to those of the control (DMSO) treatment. Values are presented as mean ± SD (n = 6 biologically independent samples examined over 20–25 stomata per leaf disc).

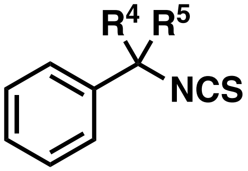

S1, R^4 = $-CH_3$ (*R*), R^5 = -H IC_{50} = **22.8**

S2, R^4 = $-CH_3$ (*S*), R^5 = -H IC_{50} = **20.4**

S3, R^4 = $-CH_2OCH_3$ (*R*), R^5 = -H IC_{50} = **7.4**

S4, R^4 = $-CH_2OCH_3$ (*S*), R^5 = -H IC_{50} = **7.7**

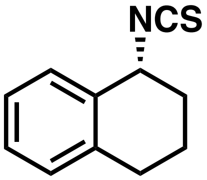

S5 IC_{50} = **72.5**

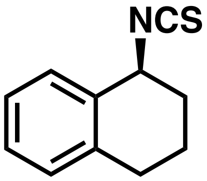

S6 IC_{50} = **69.9**

(μM)

Supplementary Fig. 5. Activities of enantiomer derivatives of BITC.

Dose-dependent inhibition of *C. benghalensis* stomatal opening was investigated and the IC_{50} values of the derivatives were calculated as done in Fig. 3.

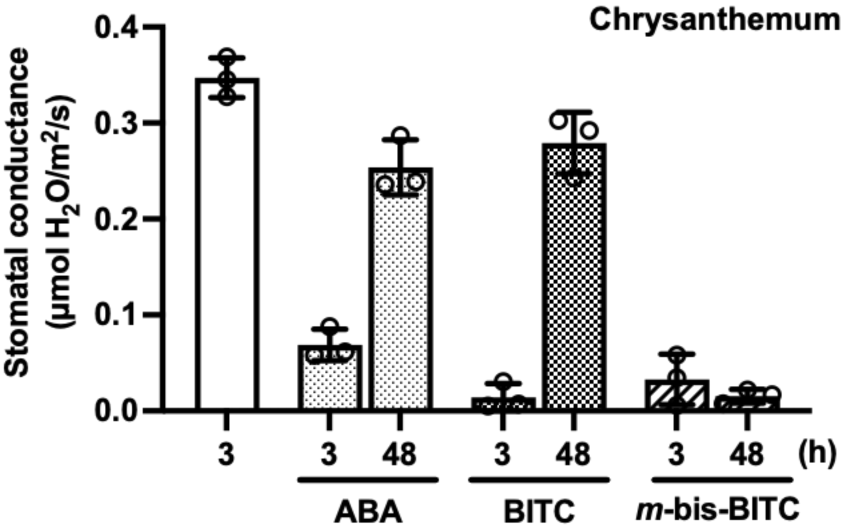

Supplementary Fig. 6. Long-term effect of ABA, BITC, and *m*-bis-BITC on stomatal conductance in Chrysanthemum leaf in a bouquet.

The leaves were treated as in Fig. 6D and the stomatal conductance was measured. Values are presented as mean ± SD (n = 3 biologically independent samples examined over two measurements per leaf).

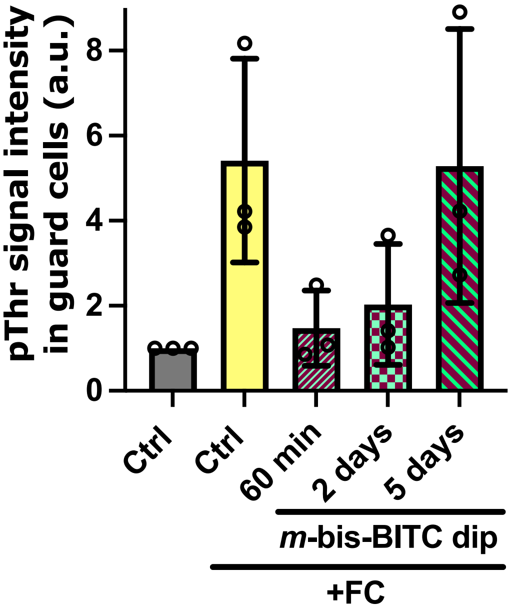

Supplementary Fig. 7. The time course of the reversion in the inhibition of PM H^+-ATPase phosphorylation by *m*-bis-BITC.

Four- to five-week-old Arabidopsis plants were dipped in DMSO (Ctrl) or 50 µM *m*-bis-BITC in 0.02% Makupica (a spreading agent) and incubated for 60 min, 2 days, or 5 days under white light (100 µmol m^{-2} s^{-1}) with a photoperiod of 16-h light/8-h dark. The leaves were then sampled and treated with DMSO or 10 µM FC (+*FC*), followed by immunohistochemical staining for phosphorylated PM H^+-ATPase in guard cells as shown in Fig. 2A. Values are presented as mean ± SD (n = 3; 50 stomata in each experiment)

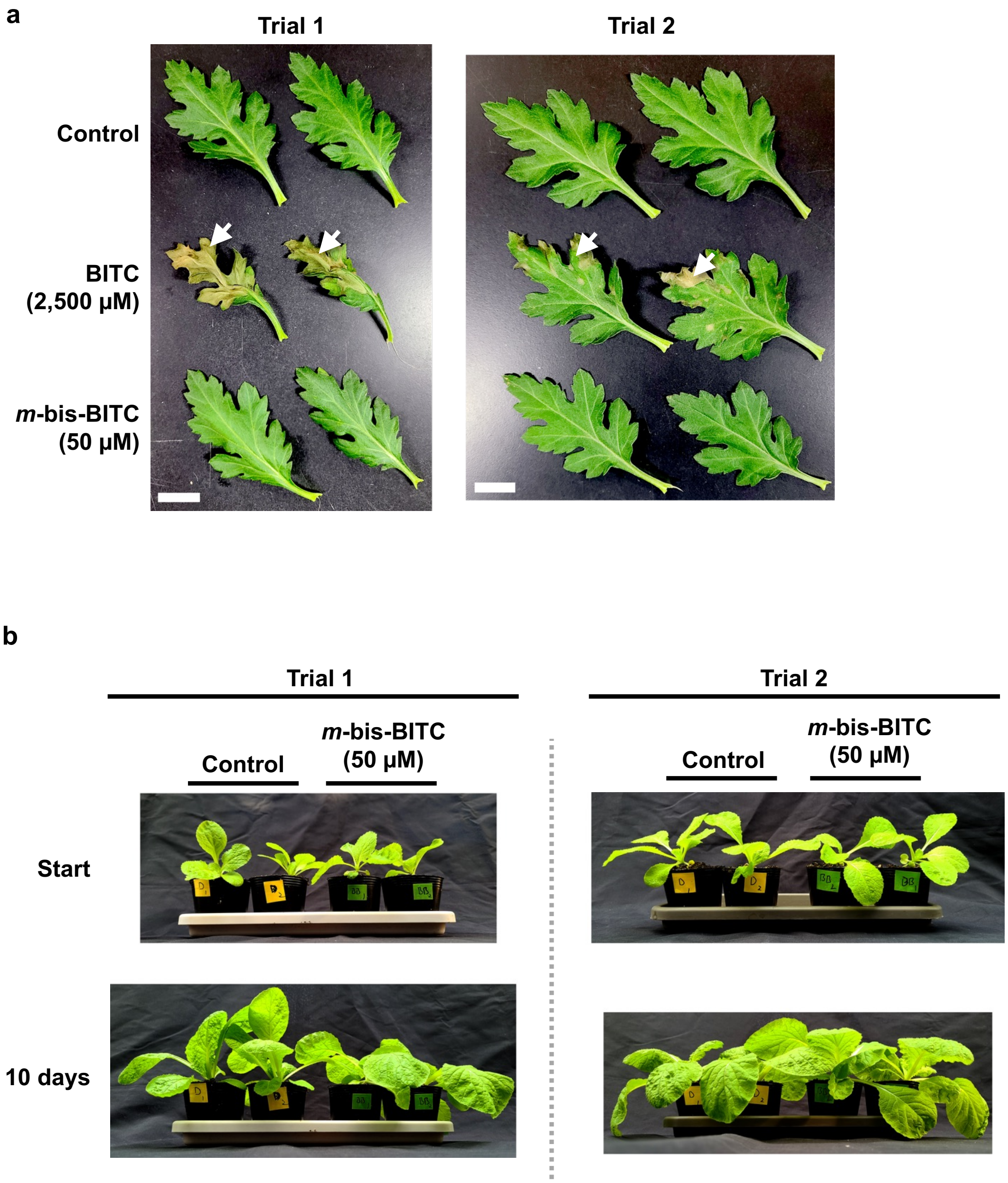

Supplementary Fig. 8. Long-term toxicity of BITCs in intact plant leaves.
(**a**) The effect of BITCs on Chrysanthemum. A bouquet of intact Chrysanthemum leaves was dipped with 2,500 µM BITC, 50 µM *m*-bis-BITC, or the same volume of DMSO (Control) and incubated at 22˚C under a photoperiod of 16-h white light (100 µmol m^{-2} s^{-1})/8-h dark. Two leaves from different bouquets were excised and photographed after 3 days. Two independent trials are displayed, showing the leaf withering (damaged area). Bars = 2 cm. (**b**) The effect of *m*-bis-BITC on *B. rapa*. Plants were treated with DMSO (Control) or 50 µM *m*-bis-BITC and incubated in a greenhouse for 10 days. Two independent trials are displayed, showing normal growth after both treatments.

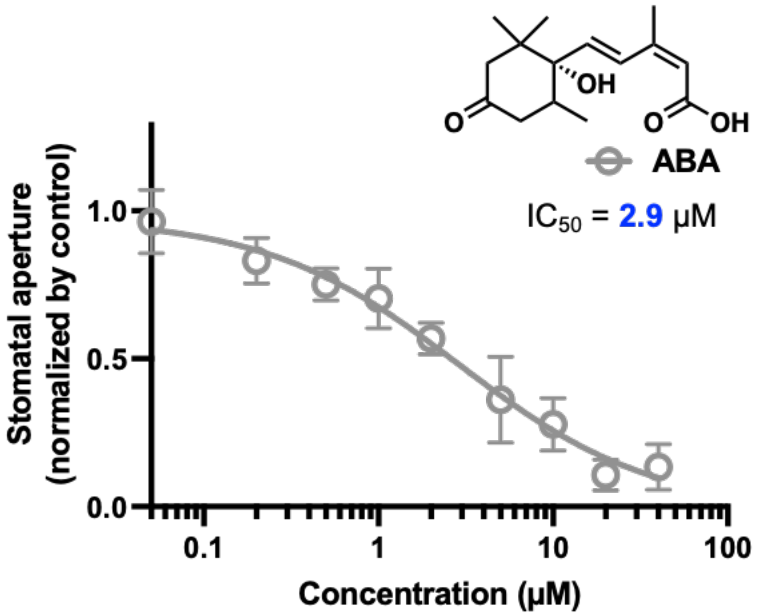

Supplementary Fig. 9. Dose-dependent effect of ABA on light-induced stomatal opening in *C. benghalensis.*

ABA was tested for its ability to inhibit stomatal opening in *C. benghalensis*, as shown in Figure 1B. Values are compared to those of the control (DMSO) treatment. Values are presented as mean ± SD (n = 6 biologically independent samples examined over 20–25 stomata per leaf disc).

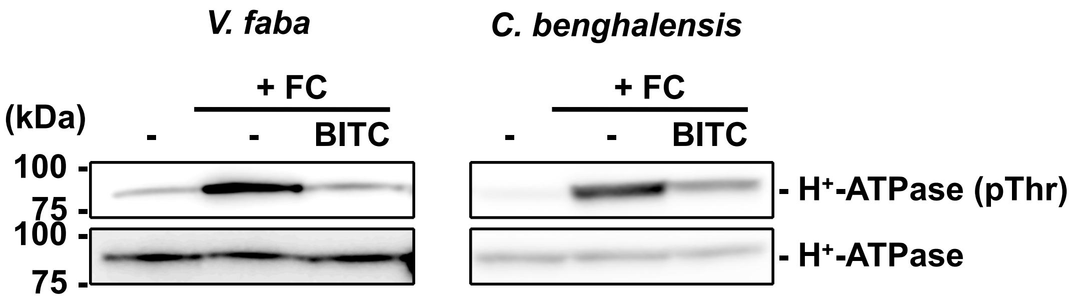

Supplementary Fig. 10. Effect of BITC on PM H^+-ATPase phosphorylation in *V. faba* or *C. benghalensis* leaf discs.

The experiment was carried out in the same manner as in Figure 2B, except that the plant samples used were leaf discs (4 mm diameter) infiltrated with DMSO (-) or 100 µM BITC 20 min before being treated with 10 µM FC for 30 min. A representative data set from a thrice-replicated experiment with different biological samples are shown.

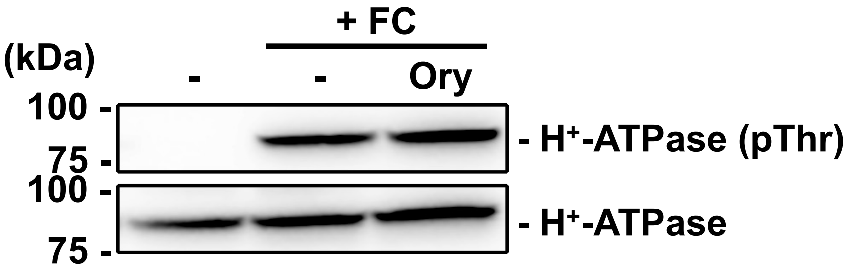

Supplementary Fig. 11. Effect of 20 μM oryzalin (Ory, Fujifilm-Wako) on the phosphorylation of PM H^+-ATPase in *A. thaliana* MCP.

As shown in Figure 2b, the experiment was carried out with a representative data set of an experiment replicated three times with different biological samples. Note that 20-min (10-μM) treatment with oryzalin is considered sufficient to disrupt microtubule filaments in Arabidopsis[1].

Supplementary Movie 1 (separate file). Real-time observation of the effect of BITC and m-bis-BITC on Chrysanthemum leaf wilting.

Experimental conditions are the same as in Figure 5C. The movie was recorded at 60x speed for 1.5 h real-time.

Supplementary Table. 1. The primer list for RT-PCR in Supplementary Fig. S3b.

**Primer Names**	**Sequence**
RAB18_fw	TGTAACGCAGTCGCATTCG
RAB18_rv	CACATCGCAGGACGTACATACAT
RD29_fw	CGAGCAAGACCCAGAAGTTCAC
RD29_rv	TTACCCGTTACACCACCTCTCA
TUB2_fw	AAACTCACTACCCCCAGCTTTG
TUB2_rv	CACCAGACATAGTAGCAGAAATCAAGT

Supplementary Methods

General Experimental Details

Unless otherwise noted, all reactants or reagents including dry solvents were obtained from commercial suppliers and used as received. 3-Methylbenzyl bromide, 4-(aminomethyl)-1-*N*-Boc-aniline, and 4-phenylbenzylamine, were purchased from Aldrich. Piperonylamine, α-bromo-*o*-xylene, 4-*tert*-butylbenzylamine, 4-butylbenzyl bromide, 4-(aminomethyl)benzonitrile hydrochloride, methyl 4-(bromomethyl)benzoate, α-bromodiphenylmethane, *p*-xylylenediamine, *m*-xylylenediamine, and 1,3,5-tris(bromomethyl)benzene were purchased from TCI. KSCN, NaI, 2-chlorobenzylamine, 2-phenylbenzylamine, 3-(bromomethyl)biphenyl, *p*-(trifluoromethoxy)benzylamine, were purchased from FUJIFILM Wako Pure Chemical Corporation. Di(1*H*-imidazol-1-yl)methanethione was purchased from Fluka chemika. Anhydrous dimethylformamide was purchased from Kanto chemical. All work-up and purification procedures were carried out with reagent-grade solvents. Analytical thin-layer chromatography (TLC) was performed using Chem Scene HPTLC Silica Gel 60 GF254. Flash column chromatography was performed with Kanto Silica Gel 60 N (spherical, neutral) (40–50 μm). Silica-gel column chromatography was performed on an Isolera Spektra instrument equipped with a Biotage SNAP Ultra 25 g cartridge. Preparative recycling gel permeation chromatography (GPC) was performed with a JAI LC-9260 II NEXT instrument equipped with JAIGEL-2HR columns using chloroform as an eluent. High-resolution mass spectra were recorded on the Thermo Fisher Scientific Exactive Plus (ESI-orbitrap)Nuclear magnetic resonance (NMR) spectra were recorded on a JNM-ECX-500 (^{1}H 500 MHz, ^{13}C 126 MHz) spectrometer. Chemical shifts for ^{1}H NMR are expressed in parts per million (ppm) relative to tetramethylsilane (δ 0.00 ppm). Chemical shifts for ^{13}C NMR are expressed in ppm relative to $CDCl_3$ (δ 77.16 ppm). Data are reported as follows: chemical shift, multiplicity (s = singlet, d = doublet, dd = doublet of doublets, t = triplet, td = triplet of doublets, q = quartet, m = multiplet), coupling constant (Hz), and integration.

General Procedures

Method A

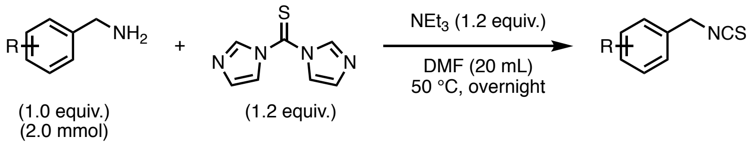

To a dried round bottom flask with a stirring bar were added di(1*H*-imidazol-1-yl)methanethione (2.4 mmol, 1.2 equiv.) and DMF (20 mL). Et_3N (2.4 mmol, 1.2 equiv.) and benzyl amine (2.0 mmol, 1.0 equiv.) were added to the flask and the mixture was stirred at 50 °C overnight. The reaction mixture was quenched by H_2O (20 mL) and extracted with Hexane/EtOAc = 1 : 1 (20 mL × 3). The organic layer was dried by Na_2SO_4 and filtered and concentrated. The crude product was purified by chromatography on silica gel to provide the desired isothiocyanate.

Method B

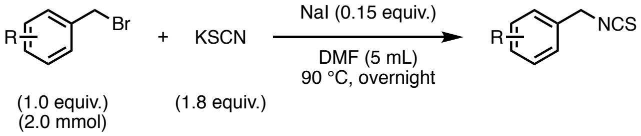

To a dried round bottom flask with a stirring bar was added potassium thiocyanate (3.6 mmol, 1.8 equiv.), NaI (0.30 mmol, 0.15 equiv.) and DMF (5 mL). Benzyl bromide (2.0 mmol, 1.0 equiv.) was added to the flask and the mixture was stirred at 90 °C overnight. The reaction mixture was quenched with H_2O (10 mL) and extracted with Et_2O (10 mL × 3). The organic layer was dried by $MgSO_4$ and filtered and concentrated. The crude product was purified by chromatography on silica gel to provide the desired isothiocyanate.

Characterization Data

Compounds **1**, **2**, **3**, **4**, **6**, **8**, **9**, **10**, **11**, **12**, **22**, **32**, and **36** are commercially available, and compounds **5**[2], **7**[3], **13**[4], **14**[2], **16**[5], **17**[4], **18**[6], **20**[5], **21**[5], **24**[5], **25**[6], **26**[7], **28**[8], **30**[7], **31**[9], **33**[2], **34**[3], **35**[3], **S1**[11], **S2**[11], **S5**[12], **S6**[12] are known compounds and showed the identical spectra according to the literature.

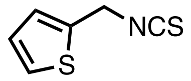

2-(isothiocyanatomethyl)thiophene (**5**): The title compound **5** was synthesized according to General Procedure (**Method A**) 150 mg, 0.97 mmol, 48% as a yellow oil.

^{1}H NMR (500 MHz, $CDCl_3$) δ 4.85 (s, 2H), 6.99 (dd, *J* = 5.0, 4.0 Hz, 1H), 7.04–7.06 (m, 1H), 7.31 (dd, *J* = 5.0, 1.0 Hz, 1H); ^{13}C NMR (126 MHz, $CDCl_3$) δ 43.85, 126.25, 126.71, 127.13, 134.24, 136.53.

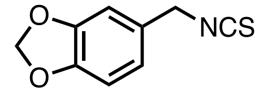

5-(Isothiocyanatomethyl)-1,3-benzodioxole (**7**): The title compound **7** was synthesized according to General Procedure (**Method A**) 197 mg, 1.02 mmol, 51% as a white solid.

^{1}H NMR (500 MHz, $CDCl_3$) δ 4.59 (s, 2H), 5.97 (s, 2H), 6.74–6.79 (m, 3H); ^{13}C NMR (126 MHz, $CDCl_3$) δ 48.63, 101.44, 107.63, 108.50, 120.66, 127.97, 132.23, 147.74, 148.18.

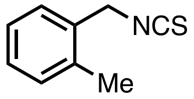

1-(Isothiocyanatomethyl)-2-methylbenzene (**13**): The title compound **13** was synthesized according to General Procedure (**Method B**) 174 mg, 1.07 mmol, 54% as pale yellow oil.

^{1}H NMR (500 MHz, $CDCl_3$) δ 2.35 (s, 3H), 4.69 (s, 2H), 7.20–7.32 (m, 4H); ^{13}C NMR (126 MHz, $CDCl_3$) δ 18.99, 47.28, 126.66, 128.02, 128.85, 130.83, 131.89, 132.45, 135.89.

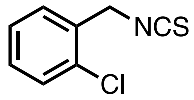

1-chloro-2-(isothiocyanatomethyl)benzene (**14**): The title compound **14** was synthesized according to General Procedure (**Method A**) 284 mg, 1.55 mmol, 78% as pale yellow oil.

^{1}H NMR (500 MHz, $CDCl_3$) δ 4.79 (s, 2H), 7.24–7.32 (m, 2H), 7.36–7.39 (m, 1H), 7.41–7.44 (m, 1H); ^{13}C NMR (126 MHz, $CDCl_3$) δ 46.75, 127.39, 128.71, 129.75, 129.79, 132.04, 132.66, 133.30.

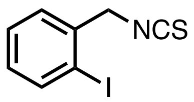

1-iodo-2-(isothiocyanatomethyl)benzene (**15**): The title compound **15** was synthesized according to General Procedure (**Method B**) 327 mg, 1.19 mmol, 60% as a colorless oil.

^{1}H NMR (500 MHz, $CDCl_3$) δ 4.77 (s, 2H), 7.06 (td, *J* = 7.5, 1.5 Hz, 1H), 7.39–7.46 (m, 2H), 7.86 (d, *J* = 8.0 Hz, 1H); ^{13}C NMR (126 MHz, $CDCl_3$) δ 53.92, 97.40, 128.38, 128.85, 130.08, 133.20, 136.65, 139.64; HRMS (ESI-positive) (*m*/*z*): $[M + Na]^+$ calcd for C_8H_6INSNa, 297.9158; found, 297.9159.

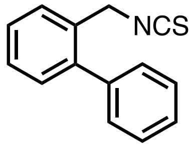

2-Phenylbenzyl isothiocyanate (**16**): The title compound **16** was synthesized according to General Procedure (**Method A**) 328 mg, 1.46 mmol, 73% as a colorless oil.

^{1}H NMR (500 MHz, $CDCl_3$) δ 4.62 (s, 2H), 7.28–7.31 (m, 3H), 7.38–7.47 (m, 5H), 7.51–7.53 (m, 1H); ^{13}C NMR (126 MHz, $CDCl_3$) δ 47.10, 127.79, 128.16, 128.37, 128.56, 128.63, 128.97, 130.40, 131.72, 131.83, 139.77, 141.36.

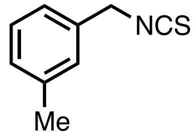

1-(Isothiocyanatomethyl)-3-methylbenzene (**17**): The title compound **17** was synthesized according to General Procedure (**Method B**) 79.5 mg, 0.487 mmol, 24% as pale yellow oil.

^{1}H NMR (500 MHz, $CDCl_3$) δ 2.37 (s, 3H), 4.68 (s, 2H), 7.10–7.12 (m, 2H), 7.15 (d, *J* = 8.0 Hz, 1H), 7.26–7.29 (m, 1H); ^{13}C NMR (126 MHz, $CDCl_3$) δ 21.54, 48.75, 124.03, 127.69, 128.99, 129.24, 131.88, 134.20, 138.94.

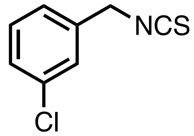

1-chloro-3-(isothiocyanatomethyl)benzene (**18**): The title compound **18** was synthesized according to General Procedure (**Method B**) 248 mg, 1.35 mmol, 68% as pale yellow oil.

^{1}H NMR (500 MHz, $CDCl_3$) δ 4.71 (s, 2H), 7.19–7.23 (m, 1H), 7.30–7.34 (m, 3H); ^{13}C NMR (126 MHz, $CDCl_3$) δ 48.08, 124.96, 126.97, 128.53, 130.28, 133.06, 134.74, 136.15.

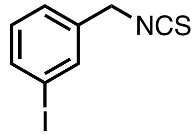

1-iodo-3-(isothiocyanatomethyl)benzene (**19**): The title compound **19** was synthesized according to General Procedure (**Method B**) 175 mg, 0.636 mmol, 32% as pale yellow oil.

^{1}H NMR (500 MHz, $CDCl_3$) δ 4.68 (s, 2H), 7.11–7.15 (m, 1H), 7.30 (d, J = 8.0 Hz, 1H), 7.66–7.70 (m, 2H); ^{13}C NMR (126 MHz, $CDCl_3$) δ 47.92, 94.67, 126.12, 130.70, 133.20, 135.82, 136.41, 137.50.; HRMS (ESI-positive) (m/z): $[M + Na]^+$ calcd for C_8H_6INSNa, 297.9158; found, 297.9160.

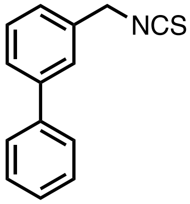

3-(Isothiocyanatomethyl)-1,1'-biphenyl (**20**) To a dried round bottom flask with a stirring bar was added potassium thiocyanate (4.0 mmol, 2.0 equiv.), NaI (0.60 mmol, 0.30 equiv.) and DMF (5 mL). 3-(bromomethyl)-1,1'-biphenyl (2.0 mmol, 1.0 equiv.) was added to the flask by stirring overnight at 90 °C. The reaction mixture was quenched with H_2O (10 mL) and extracted with Et_2O (10 mL × 3). The organic layer was dried by $MgSO_4$ and filtered and concentrated. The crude product was purified by chromatography on silica gel provided the title compound **20 (**193 mg, 0.858 mmol 43%) product as pale pink oil.

^{1}H NMR (500 MHz, $CDCl_3$) δ 4.77 (s, 2H), 7.29 (d, J = 7.5 Hz, 1H), 7.36–7.39 (m, 1H), 7.44–7.47 (m, 3H), 7.51 (s, 1H), 7.55–7.60 (m, 3H); ^{13}C NMR (126 MHz, $CDCl_3$) δ 48.83, 125.75, 125.78, 127.30, 127.82, 129.02, 129.55, 132.52, 134.88, 140.50, 142.18 (one sp^2 signal was not observed because of overlapping).

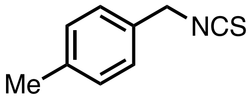

1-(isothiocyanatomethyl)-4-methylbenzene (**21**): The title compound **21** was synthesized according to General Procedure (**Method A**) 198 mg, 1.21 mmol, 61% as pale yellow oil.

^{1}H NMR (500 MHz, $CDCl_3$) δ 2.36 (s, 3H), 4.67 (s, 2H), 7.16–7.23 (m, 4H); ^{13}C NMR (126 MHz, $CDCl_3$) δ 21.28, 48.62, 127.00, 129.75, 131.34, 131.95, 138.38.

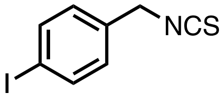

1-iodo-4-(isothiocyanatomethyl)benzene (**23**): The title compound **23** was synthesized according to General Procedure (**Method B**) 354 mg, 1.29 mmol, 64% as pale yellow oil.

^{1}H NMR (500 MHz, $CDCl_3$) δ 4.65 (s, 2H), 7.05 (d, J = 8.0 Hz, 2H), 7.70 (d, J = 8.0 Hz, 2H); ^{13}C NMR (126 MHz, $CDCl_3$) δ 48.30, 94.05, 128.76, 133.04, 133.97, 138.09; HRMS (ESI-positive) (m/z): $[M + Na]^+$ calcd for C_8H_6INSNa, 297.9158; found, 297.9161.

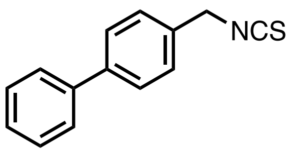

4-(Isothiocyanatomethyl)-1,1'-biphenyl (24): The title compound **24** was synthesized according to General Procedure (**Method A**) 222 mg, 1.07 mmol, 49% as a white solid.

^{1}H NMR (500 MHz, $CDCl_3$) δ 4.76 (s, 2H), 7.35–7.40 (m, 3H), 7.43–7.47 (m, 2H), 7.57–7.63 (m, 4H); ^{13}C NMR (126 MHz, $CDCl_3$) δ 48.45, 127.13, 127.37, 127.68, 128.93, 132.33, 133.22, 140.31, 141.35 (one sp^2 signal was not observed because of overlapping).

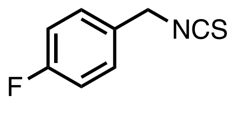

1-fluoro-4-(isothiocyanatomethyl)benzene (**25**) To a dried round bottom flask with a stirring bar was added potassium thiocyanate (3.6 mmol, 1.8 equiv.), NaI (0.60 mmol, 0.30 equiv.) and DMF (5 mL). 1-(bromomethyl)-4-fluorobenzene (2.0 mmol, 1.0 equiv.) was added to the flask and the mixture was stirred at 90 °C overnight. The reaction mixture was quenched with H_2O (10 mL) and extracted with Et_2O (10 mL × 3). The organic layer was dried by $MgSO_4$ and filtered and concentrated. The crude product was purified by chromatography on silica gel and preparative recycling gel permeation chromatography provided the title compound **25** (89.2 mg, 0.533 mmol 27%) product as a colorless oil.

^{1}H NMR (500 MHz, $CDCl_3$) δ 4.68 (s, 2H), 7.06–7.10 (m, 2H), 7.29 (dd, J = 8.5, 5.0 Hz, 2H); ^{13}C NMR (126 MHz, $CDCl_3$) δ 48.17, 116.06 (d, J = 21.7 Hz), 128.85 (d, J = 8.3 Hz), 130.19 (d, J = 3.7 Hz), 132.86, 162.70 (d, J = 248.7 Hz).

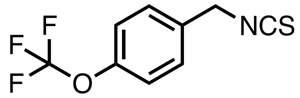

4-(Trifluoromethoxy)benzyl isothiocyanate (**26**): The title compound **26** was synthesized according to General Procedure (**Method A**) 148 mg, 0.636 mmol, 32% as a colorless oil.

^{1}H NMR (500 MHz, $CDCl_3$) δ 4.74 (s, 2H), 7.25 (d, J = 8.5 Hz, 2H), 7.36 (d, J = 8.5 Hz, 2H); ^{13}C NMR (126 MHz, $CDCl_3$) δ 48.08, 120.52 (q, J = 257.2 Hz), 121.55, 128.50, 133.10, 149.19, (one sp^2 signal was not observed because of overlapping).

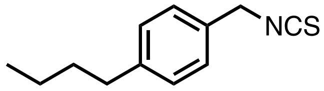

1-Butyl-4-(isothiocyanatomethyl)benzene (**27**): The title compound **27** was synthesized according to General Procedure (**Method B**) 310 mg, 1.51 mmol, 76% as a colorless oil.

^{1}H NMR (500 MHz, $CDCl_3$) δ 0.92 (t, J = 7.5 Hz, 3H), 1.34 (sextet, J = 7.5, Hz, 2H), 1.55–1.61 (m, 2H), 2.60 (t, J = 7.5 Hz, 2H), 4.65 (s, 2H), 7.17–7.23 (m, 4H); ^{13}C NMR (126 MHz, $CDCl_3$) δ 14.04, 22.39, 33.65, 35.35, 48.55, 126.91, 129.05, 131.45, 131.75, 143.31; HRMS (ESI-positive) (*m*/*z*): $[M + Na]^+$ calcd for $C_{12}H_{15}NSNa$, 228.0817; found, 228.0820.

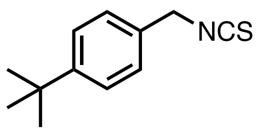

1-*tert*-Butyl-4-isothiocyanatomethylbenzene (**28**): The title compound **28** was synthesized according to General Procedure (**Method A**) 278 mg, 1.35 mmol, 68% as a white solid.

^{1}H NMR (500 MHz, $CDCl_3$) δ 1.32 (s, 9H), 4.67 (s, 2H), 7.25 (d, J = 8.0 Hz, 2H), 7.40 (d, J = 8.0 Hz, 2H); ^{13}C NMR (126 MHz, $CDCl_3$) δ 31.41, 34.75, 48.52, 126.03, 126.81, 131.37, 131.97, 151.65.

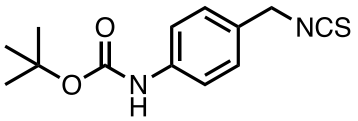

29: The title compound **29** was synthesized according to General Procedure (**Method A**) 213 mg, 0.805 mmol, 41% as a white solid.

^{1}H NMR (500 MHz, $CDCl_3$) δ 1.52 (s, 9H), 4.63 (s, 2H), 6.69 (br, 1H), 7.21 (d, J = 8.5 Hz, 2H), 7.38 (d, J = 8.5 Hz, 2H); ^{13}C NMR (126 MHz, $CDCl_3$) δ 28.37, 48.35, 80.84, 118.83, 127.80, 128.61, 132.00, 138.62, 152.73; HRMS (ESI-positive) (*m*/*z*): $[M + Na]^+$ calcd for $C_{13}H_{16}N_2O_2SNa$, 287.0825; found, 287.0824.

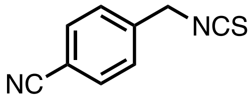

4-(Isothiocyanatomethyl)benzonitrile (**30**): To a dried round bottom flask with a stirring bar was added di(1*H*-imidazol-1-yl)methanethione (2.4 mmol, 1.2 equiv.) and DMF (20 mL). Et_3N (4.4 mmol, 2.2 equiv.) and 4-(isothiocyanatomethyl)benzonitrile hydrochloride (2.0 mmol, 1.0 equiv.) were added to the flask and the mixture was stirred at 50 °C overnight. The reaction mixture was quenched by H_2O (20 mL) and extracted with Hexane/EtOAc = 1:1 (20 mL × 3). The organic layer was dried by Na_2SO_4 and filtered and concentrated. The crude product was purified by chromatography on silica gel provided by the title compound **30** (34.6 mg, 0.199 mmol, 10%) as a pale yellow solid.

^{1}H NMR (500 MHz, $CDCl_3$) δ 4.83 (s, 2H), 7.46 (d, J = 8.5 Hz, 2H), 7.71 (d, J = 8.5 Hz, 2H); ^{13}C NMR (126 MHz, $CDCl_3$) δ 48.35, 112.47, 118.39, 127.48, 132.90, 134.46, 139.57; HRMS (ESI-positive) (m/z): $[M + Na]^+$ calcd for $C_9H_6N_2SNa$, 197.0144; found, 197.0148.

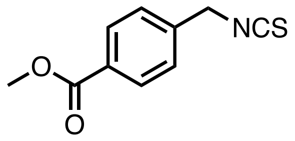

Methyl 4-(isothiocyanatomethyl)benzoate (**31**): To a dried round bottom flask with a stirring bar was added potassium thiocyanate (7.2 mmol, 3.6 equiv.), NaI (0.60 mmol, 0.30 equiv.) and DMF (5 mL). methyl 4-(bromomethyl)benzoate (2.0 mmol, 1.0 equiv.) was added to the flask and the mixture was stirred at 90 °C overnight. The reaction mixture was quenched with H_2O (10 mL) and extracted with Et_2O (10 mL × 3). The organic layer was dried by $MgSO_4$ and filtered and concentrated. The crude product was purified by chromatography on silica gel provided the title compound **31** (106 mg, 0.509 mmol 25%) product as a white solid.

^{1}H NMR (500 MHz, $CDCl_3$) δ 3.93 (s, 3H), 4.80 (s, 2H), 7.39 (d, J = 8.5 Hz, 2H), 8.06 (d, J = 8.5 Hz, 2H); ^{13}C NMR (126 MHz, $CDCl_3$) δ 48.44, 52.35, 126.74, 130.25, 130.31, 133.33, 139.20, 166.53.

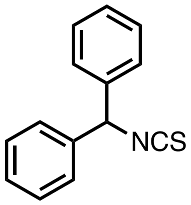

1,1'-(Isothiocyanatomethylene)bis-benzene (**33**): The title compound **33** was synthesized according to General Procedure (**Method B**) 317 mg, 1.41 mmol, 73% as a white solid.

^{1}H NMR (500 MHz, $CDCl_3$) δ 5.99 (s, 1H), 7.31–7.34 (m, 6H), 7.35–7.39 (m, 4H); ^{13}C NMR (126 MHz, $CDCl_3$) δ 64.67, 126.69, 128.43, 129.04, 134.54, 139.28.

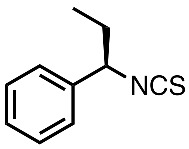

(*R*)-(1-isothiocyanatopropyl)benzene (**34**): The title compound **34** was synthesized following the general procedure (**Method A**) 333 mg, 1.88 mmol, 93% as a colorless oil.

^{1}H NMR (500 MHz, $CDCl_3$) δ 1.00 (t, J = 7.5 Hz, 3H), 1.86–2.00 (m, 2H), 4.67 (dd, J = 7.5, 6.0 Hz, 1H), 7.26–7.32 (m, 3H), 7.34–7.38 (m, 2H); ^{13}C NMR (126 MHz, $CDCl_3$) δ 10.57, 32.37, 63.23, 125.93, 128.20, 128.82, 131.83, 138.85.

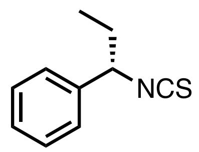

(*S*)-(1-isothiocyanatopropyl)benzene (**35**): The title compound **35** was synthesized following the general procedure (**Method A**) 288 mg, 1.63 mmol, 85% as a colorless oil.

^{1}H NMR (500 MHz, $CDCl_3$) δ 1.02 (t, J = 7.5 Hz, 3H), 1.90–2.00 (m, 2H), 4.70 (dd, J = 8.0, 6.0 Hz, 1H), 7.27–7.34 (m, 3H), 7.37–7.40 (m, 2H); ^{13}C NMR (126 MHz, $CDCl_3$) δ 10.54, 32.34, 63.22, 125.90, 128.17, 128.79, 131.86, 138.84.

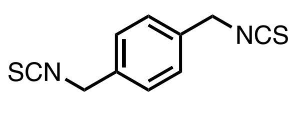

1,4-Bis(isothiocyanatomethyl)benzene (**37**): To a dried round bottom flask with a stirring bar were added di(1*H*-imidazol-1-yl)methanethione (10 mmol, 5 equiv.) and DMF (15 mL). Et_3N (4.8 mmol, 2.4 equiv.) and a DMF (5 mL) solution of 1,4-phenylenedimethanamine (2.0 mmol, 1.0 equiv.) were added to the flask and the mixture was stirred at 50 °C overnight. The reaction mixture was quenched by H_2O (20 mL) and extracted with Hexane/EtOAc = 1:1 (20 mL × 3). The organic layer was washed with 1 M HCl aq. and water (three times). The organic layer was then dried by Na_2SO_4 and filtered and concentrated. The crude product was purified by chromatography on silica gel provided title compound **37** (221 mg, 1.00 mmol, 50%) as a pale yellow solid.

^{1}H NMR (500 MHz, $CDCl_3$) δ 4.73 (s, 4H), 7.35 (s, 4H); ^{13}C NMR (126 MHz, $CDCl_3$) δ 48.37, 127.52, 132.83, 134.59; HRMS (ESI-positive) (*m*/*z*): $[M + Na]^+$ calcd for $C_{10}H_8N_2S_2Na$, 243.0021; found, 243.0022.

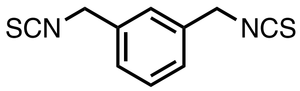

1,3-Bis(isothiocyanatomethyl)benzene (**38**) To a dried round bottom flask with a stirring bar were added di(1*H*-imidazol-1-yl)methanethione (10 mmol, 5 equiv.) and DMF (15 mL). Et_3N (4.8 mmol, 2.4 equiv.) and a DMF (5 mL) solution of 1,3-phenylenedimethanamine (2.0 mmol, 1.0 equiv.) were added to the flask and the mixture was stirred at 50 °C overnight. The reaction mixture was quenched by H_2O (20 mL) and extracted with EtOAc (20 mL × 3). The organic layer was washed with 1 M HCl aq. and brine (three times). The organic layer was then dried by Na_2SO_4 and filtered and concentrated. The crude product was purified by chromatography on silica gel provided title compound **38** (306 mg, 1.39 mmol, 69%) as a pale yellow solid.

^{1}H NMR (500 MHz, $CDCl_3$) δ 4.74 (s, 4H), 7.25 (s, 1H), 7.30 (d, J = 7.5 Hz, 2H), 7.42 (t, J = 7.5 Hz, 1H); ^{13}C NMR (126 MHz, $CDCl_3$) δ 48.47, 125.28, 126.86, 129.71, 132.78, 135.18; HRMS (ESI-positive) (*m*/*z*): $[M + Na]^+$ calcd for $C_{10}H_8N_2S_2Na$, 243.0021; found, 243.0024.

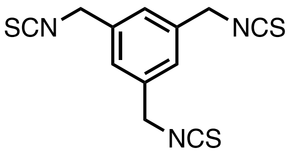

1,3,5-Tris(isothiocyanatomethyl)benzene (**39**): To a dried 100 ml round bottom flask with a stirring bar were added KSCN (14 mmol, 10 equiv.), NaI (6 mmol, 4.3 equiv.), and 1,3,5-tris(bromomethyl)benzene (1.4 mmol, 1.0 equiv.). Then, DMF (10 mL) was added to the flask and the mixture was stirred at 90 °C for 24 h. The reaction mixture was diluted with H_2O (20 mL) and extracted with diethyl ether (3 × 20 mL). The organic layer was dried over $MgSO_4$, then it was concentrated under reduced pressure. The crude product was purified by flash column chromatography on silica gel and preparative recycling gel permeation chromatography afforded title compound **39** (137 mg, 0.47 mmol, 34%) as a white solid.

^{1}H NMR (500 MHz, $CDCl_3$) δ 4.79 (s, 6H), 7.26 (s, 3H); ^{13}C NMR (126 MHz, $CDCl_3$) δ 48.38, 125.29, 133.89, 136.39; HRMS (ESI-positive) (*m*/*z*): $[M + Na]^+$ calcd for $C_{12}H_9N_3S_3Na$, 313.9851; found, 313.9848.

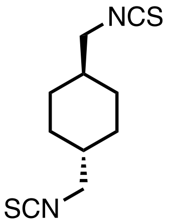

***trans*-1,4-bis(isothiocyanatomethyl)cyclohexane** (**40**): To a dry round bottom flask with a stirring bar were added di(1*H*-imidazol-1-yl)methanethione (10 mmol, 5 equiv.) and DMF (15 mL). Et_3N (9.6 mmol, 4.8 equiv.) and a DMF (5 mL) solution of *trans*-1,4-Bis(aminomethyl)cyclohexane (2.0 mmol, 1.0 equiv.) were added to the flask and the mixture was stirred overnight at 50 °C. The reaction mixture was quenched by H_2O (20 mL) and extracted with EtOAc (20 mL × 3). The organic layer was washed with 1 M HCl aq. and brine (three times). The organic layer was then dried by Na_2SO_4, filtered, and concentrated. The crude product was purified by chromatography on silica gel, which provided title compound **40** (315 mg, 1.39 mmol, 70%) as a white solid.

^{1}H NMR (500 MHz, $CDCl_3$) δ 1.07–1.16 (m, 4H), 1.60–1.72 (m, 2H), 1.85–1.93 (m, 4H), 3.41 (d, $J = 6.5$ Hz, 4H); ^{13}C NMR (126 MHz, $CDCl_3$) δ 29.39, 38.17, 50.83, 129.92.; HRMS (ESI-positive) (*m*/*z*): $[M + Na]^+$ calcd for $C_{10}H_{14}N_2S_2Na$, 249.0491; found, 249.0491.

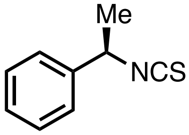

(*R*)-(1-isothiocyanatoethyl)benzene (**S1**): The title compound **S1** was synthesized according to the general procedure (**Method A**) 333 mg, 2.04 mmol, quantitative yield as a colorless oil.

^{1}H NMR (500 MHz, $CDCl_3$) δ 1.60 (d, *J* = 7.0 Hz, 3H), 4.85 (q, *J* = 7.0 Hz, 1H), 7.26–7.30 (m, 3H), 7.32–7.36 (m, 2H); ^{13}C NMR (126 MHz, $CDCl_3$) δ 24.92, 56.96, 125.35, 128.14, 128.84, 132.11, 140.06.

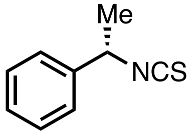

(*S*)-(1-isothiocyanatoethyl)benzene (**S2**): The title compound **S2** was synthesized according to the general procedure (**Method A**) 264 mg, 1.62 mmol, 81% as a colorless oil.

^{1}H NMR (500 MHz, $CDCl_3$) δ 1.67 (d, *J* = 7.0 Hz, 3H), 4.90 (q, *J* = 7.0 Hz, 1H), 7.29–7.34 (m, 3H), 7.36–7.40 (m, 2H); ^{13}C NMR (126 MHz, $CDCl_3$) δ 25.09, 57.12, 125.51, 128.31, 129.00, 132.24, 140.22.

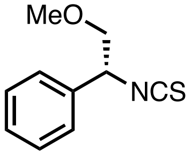

(*R*)-(1-isothiocyanato-2-methoxyethyl)benzene (**S3**): The title compound **S3** was synthesized according to the general procedure (**Method A**) (4.7 mmol scale) 592 mg, 3.05 mmol, 65% as a colorless oil.

^{1}H NMR (500 MHz, $CDCl_3$) δ 3.42 (s, 3H), 3.60–3.65 (m, 2H), 4.96 (dd, *J* = 7.5, 5.0 Hz, 1H), 7.31–7.40 (m, 5H); ^{13}C NMR (126 MHz, $CDCl_3$) δ 59.27, 61.43, 76.88, 126.42, 128.71, 128.94, 134.97, 135.78.; HRMS (ESI-positive) (*m*/*z*): $[M + Na]^+$ calcd for $C_{10}H_{11}NOSNa$, 216.0454; found, 216.0455.

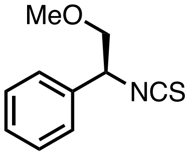

(*S*)-(1-isothiocyanato-2-methoxyethyl)benzene (**S4**): The title compound **S4** was synthesized according to the general procedure (**Method A**) (0.49 mmol scale) 49.0 mg, 0.254 mmol, 52% as a colorless oil.

^{1}H NMR (500 MHz, $CDCl_3$) δ 3.43 (s, 3H), 3.60–3.66 (m, 2H), 4.96 (dd, *J* = 7.5, 5.0 Hz, 1H), 7.32–7.41 (m, 5H); ^{13}C NMR (126 MHz, $CDCl_3$) δ 59.34, 61.50, 76.96, 126.46, 128.76, 128.99, 135.04, 135.84.; HRMS (ESI-positive) (*m*/*z*): $[M + Na]^+$ calcd for $C_{10}H_{11}NOSNa$, 216.0454; found, 216.0455.

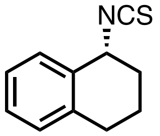

(*R*)-1-isothiocyanato-1,2,3,4-tetrahydronaphthalene (**S5**): The title compound **S5** was synthesized according to the general procedure (**Method A**) 386.9 mg, 2.04 mmol, quantitative yield as pale yellow oil.

^{1}H NMR (500 MHz, $CDCl_3$) δ 1.77–1.85 (m, 1H), 1.92–2.01 (m, 1H), 2.02–2.13 (m, 2H), 2.68–2.76 (m, 1H), 2.80–2.86 (m, 1H), 4.89 (t, *J* = 5.5 Hz, 1H), 7.09–7.12 (m, 1H), 7.17–7.24 (m, 2H), 7.31–7.34 (m, 1H); ^{13}C NMR (126 MHz, $CDCl_3$) δ 19.32, 28.57, 30.81, 55.74, 126.47, 128.33, 128.56, 129.48, 131.70, 133.18, 136.40.

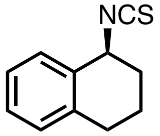

(*S*)-1-isothiocyanato-1,2,3,4-tetrahydronaphthalene (**S6**): The title compound **S6** was synthesized according to the general procedure (**Method A**) 373 mg, 1.97 mmol, 98% as pale yellow oil.

^{1}H NMR (500 MHz, $CDCl_3$) δ 1.75–1.83 (m, 1H), 1.90–1.99 (m, 1H), 2.00–2.11 (m, 2H), 2.67–2.74 (m, 1H), 2.78–2.85 (m, 1H), 4.88 (t, *J* = 5.5 Hz, 1H), 7.07–7.11 (m, 1H), 7.16–7.23 (m, 2H), 7.28–7.33 (m, 1H); ^{13}C NMR (126 MHz, $CDCl_3$) δ 19.28, 28.53, 30.77, 55.70, 126.43, 128.29, 128.51, 129.43, 131.67, 133.14, 136.36.

^{1}H and ^{13}C NMR spectra

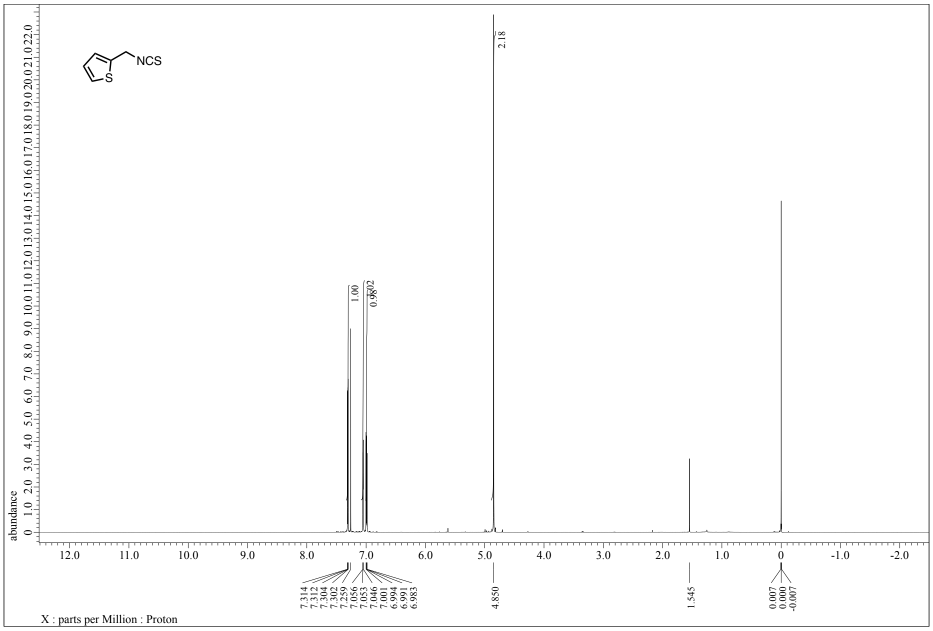

Supplementary Fig. 13: ^{1}H NMR (500 MHz) spectrum of **5**.

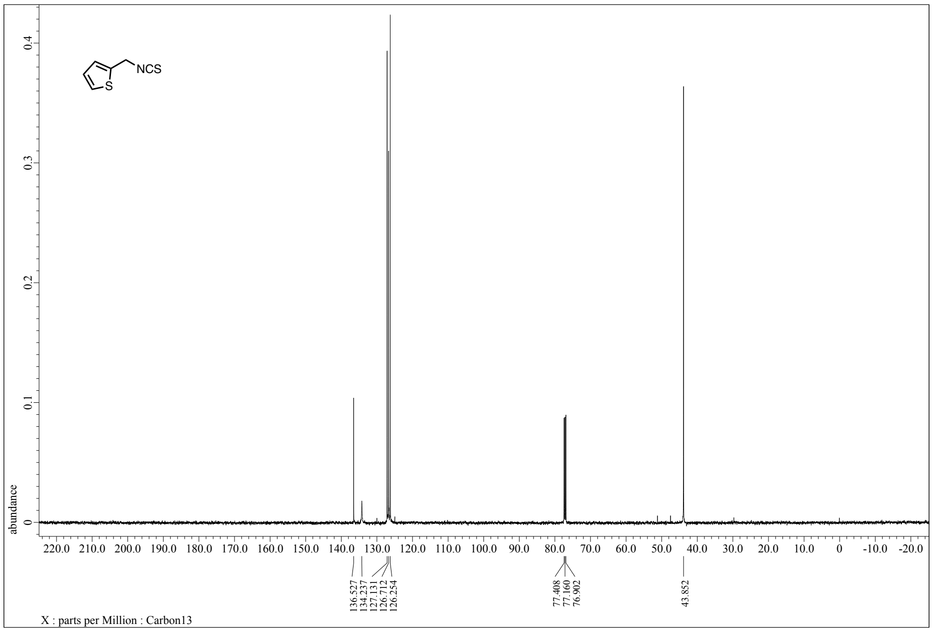

Supplementary Fig. 14: ^{13}C NMR (126 MHz) spectrum of **5**.

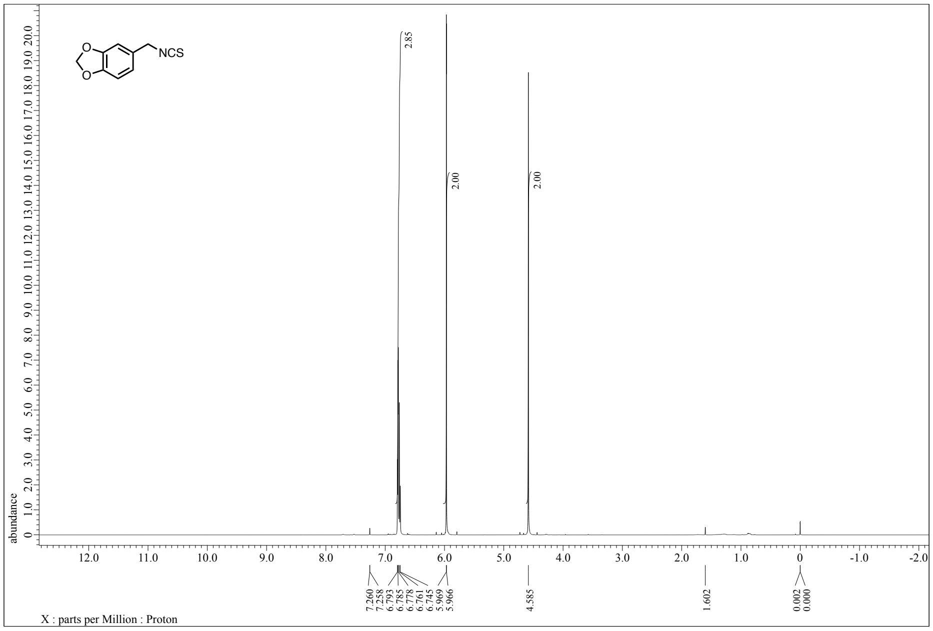

Supplementary Fig. 15: ^{1}H NMR (500 MHz) spectrum of **7**.

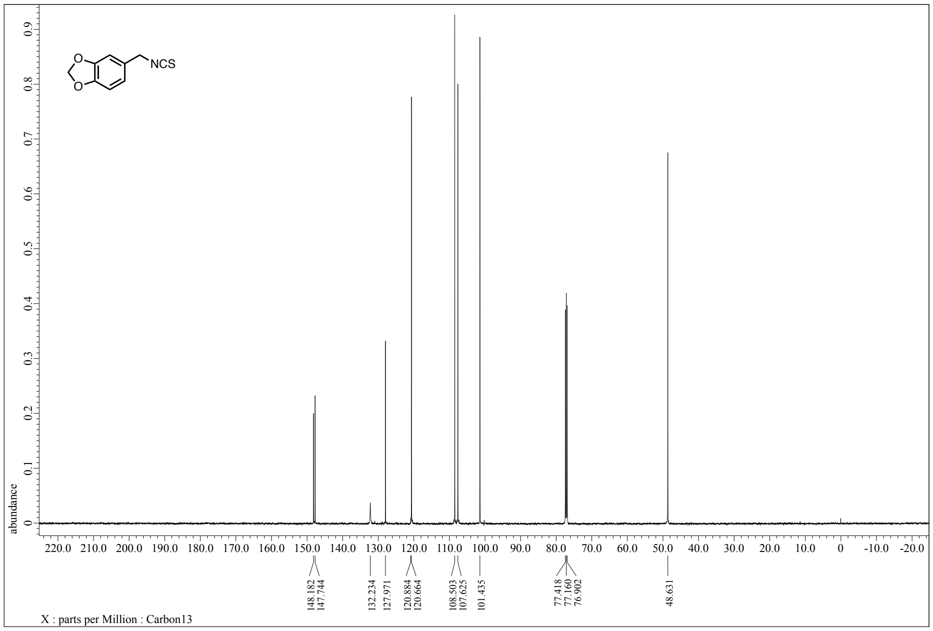

Supplementary Fig. 16: ^{13}C NMR (126 MHz) spectrum of **7**.

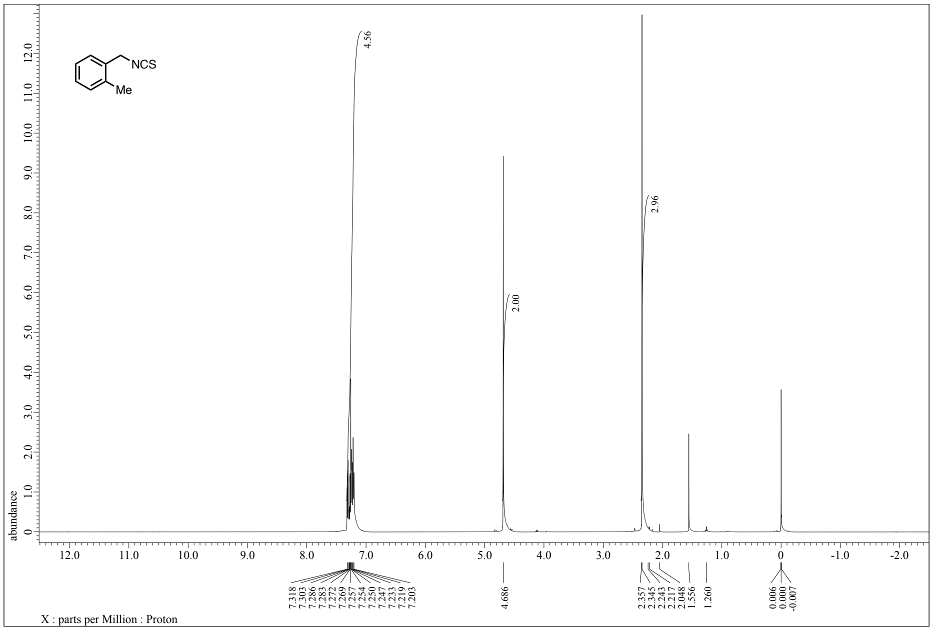

Supplementary Fig. 17: ^{1}H NMR (500 MHz) spectrum of **13**.

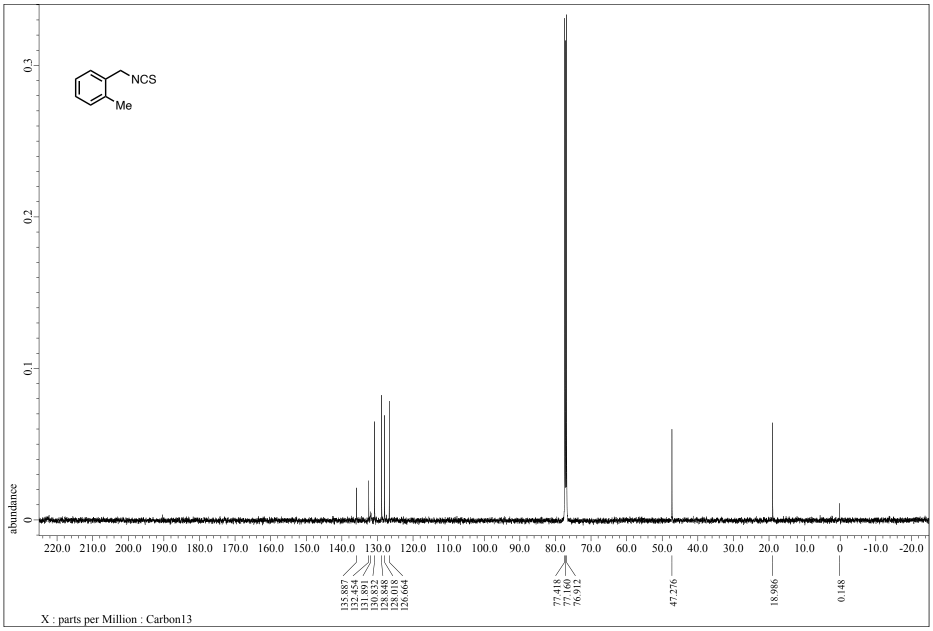

Supplementary Fig. 18: ^{13}C NMR (126 MHz) spectrum of **13**.

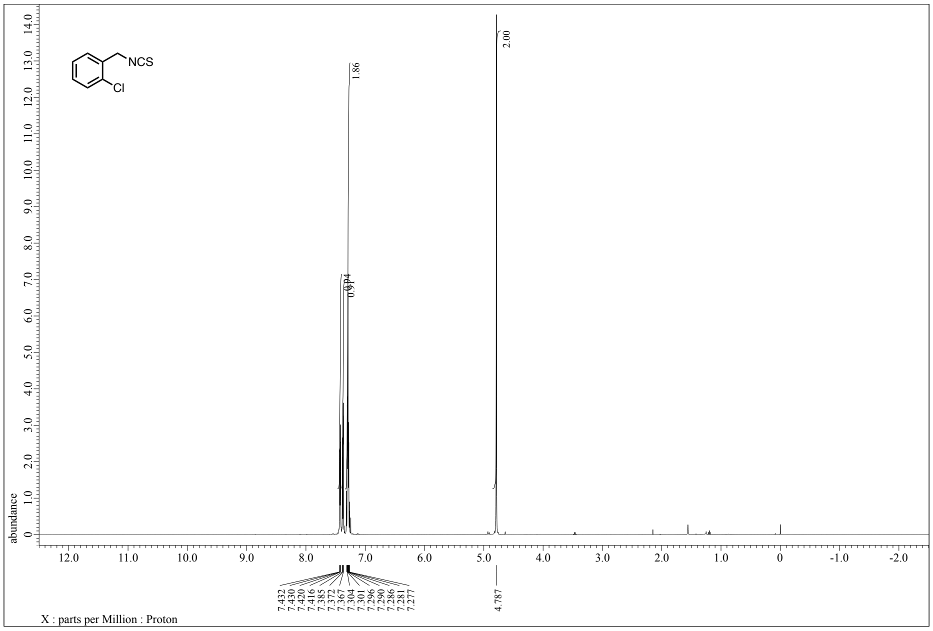

Supplementary Fig. 19: ^{1}H NMR (500 MHz) spectrum of **14**.

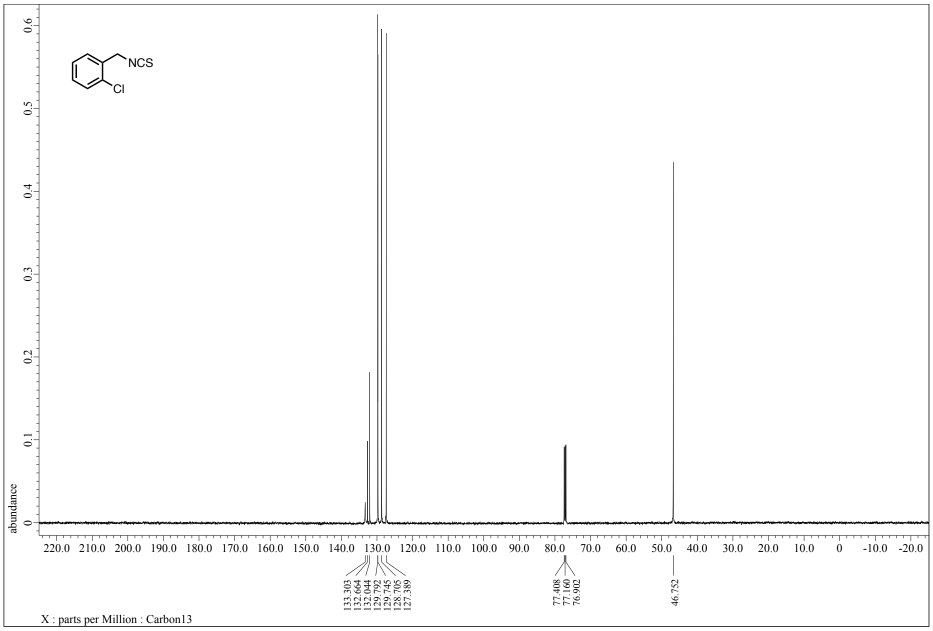

Supplementary Fig. 20: ^{13}C NMR (126 MHz) spectrum of **14**.

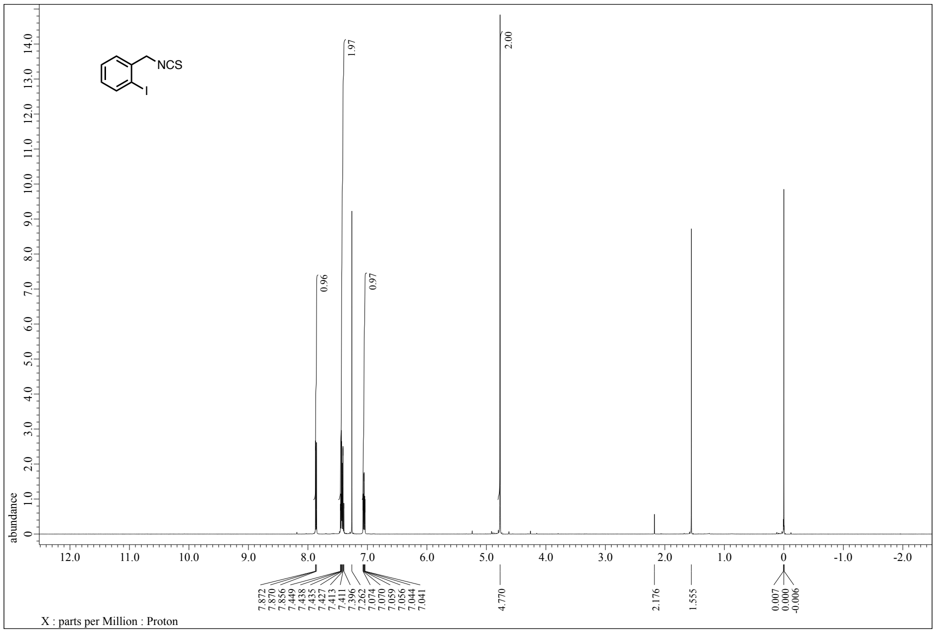

Supplementary Fig. 21: ^{1}H NMR (500 MHz) spectrum of **15**.

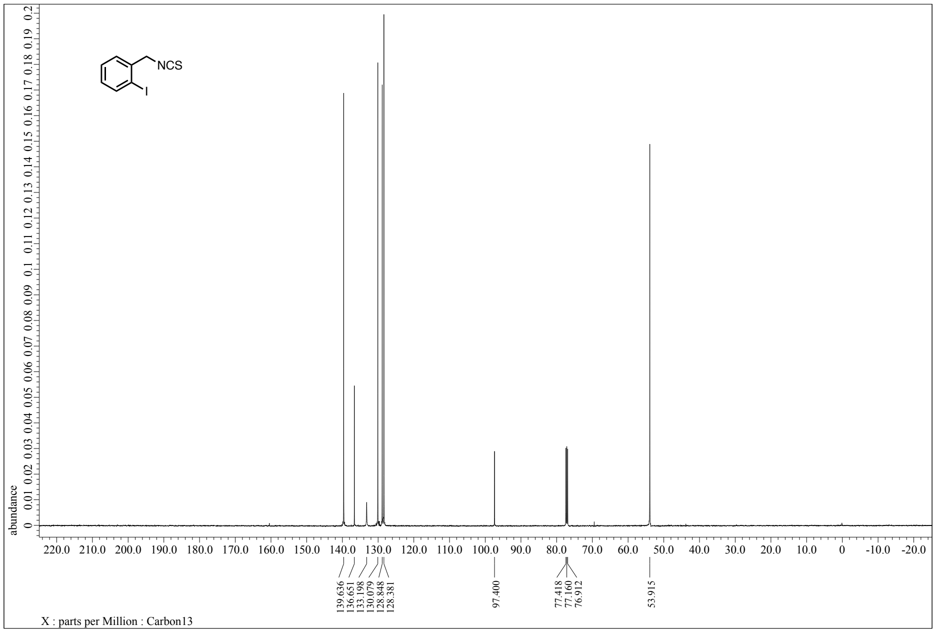

Supplementary Fig. 22: ^{13}C NMR (126 MHz) spectrum of **15**.

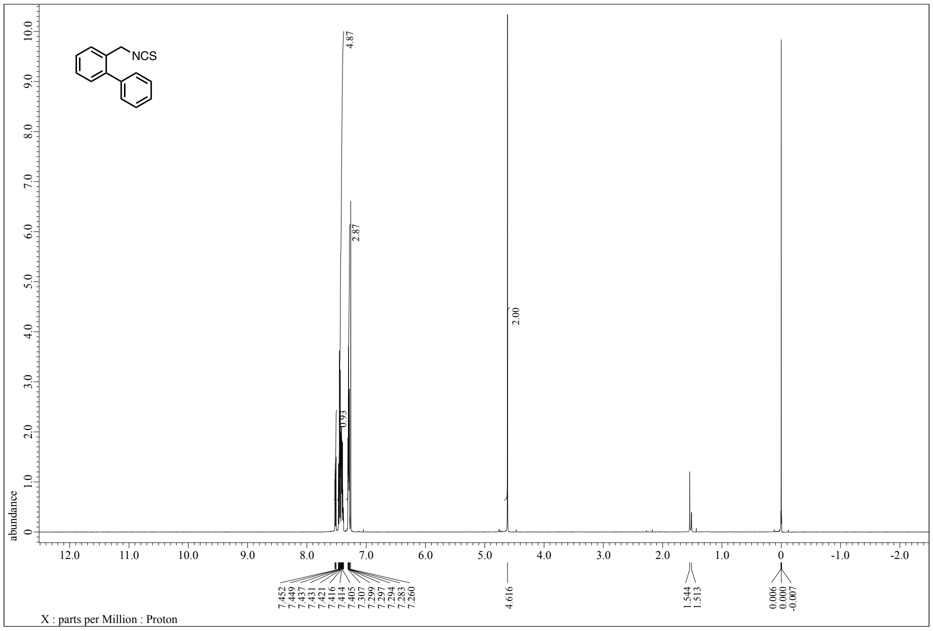

Supplementary Fig. 23: ^{1}H NMR (500 MHz) spectrum of **16**.

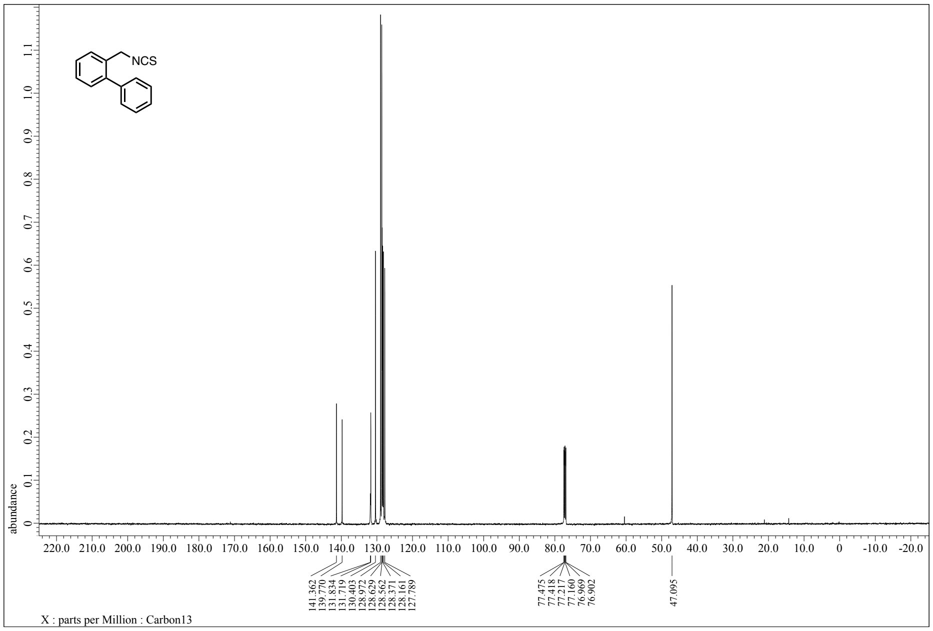

Supplementary Fig. 24: ^{13}C NMR (126 MHz) spectrum of **16**.

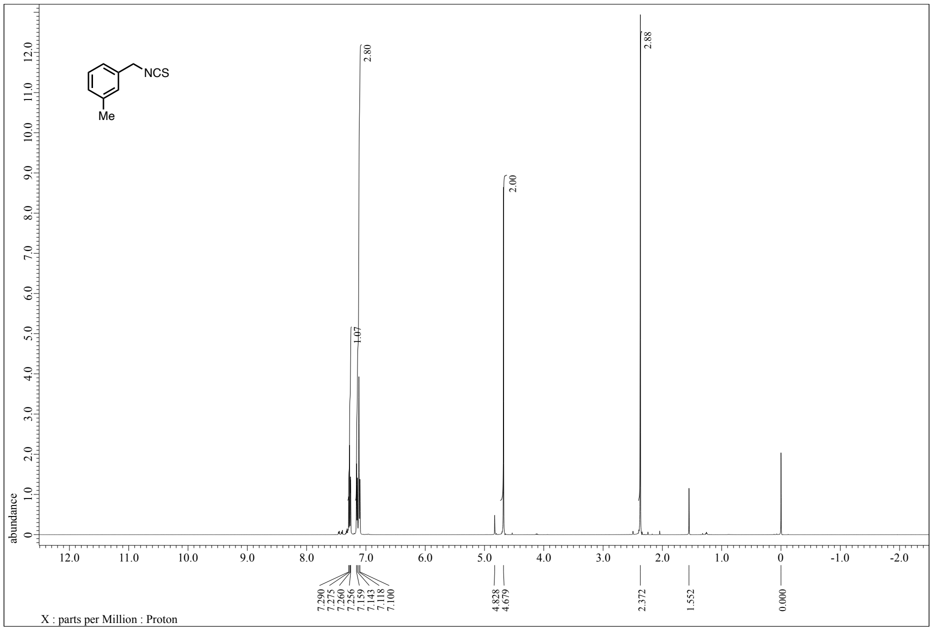

Supplementary Fig. 25: ^{1}H NMR (500 MHz) spectrum of **17**.

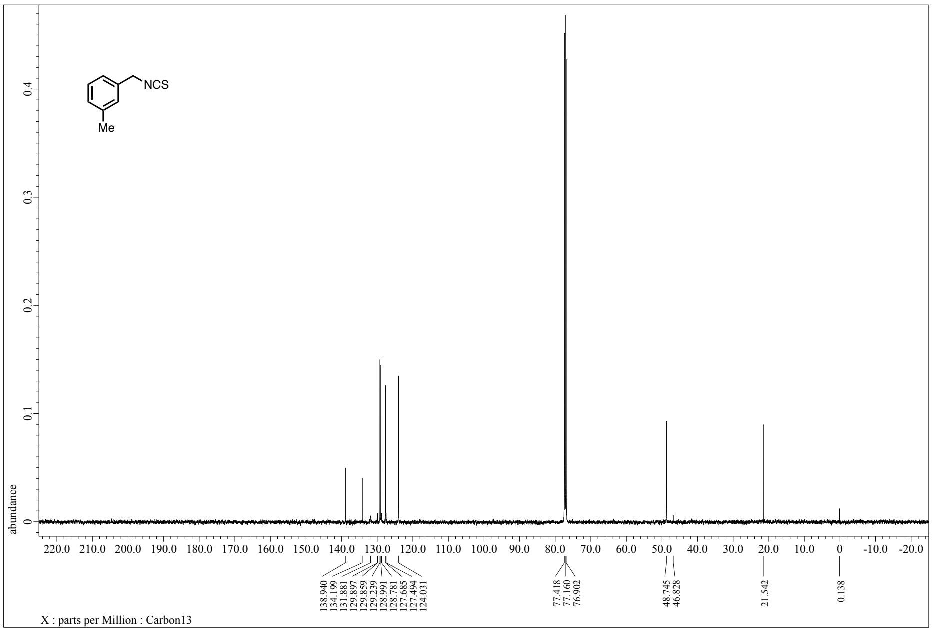

Supplementary Fig. 26: ^{13}C NMR (126 MHz) spectrum of **17**.

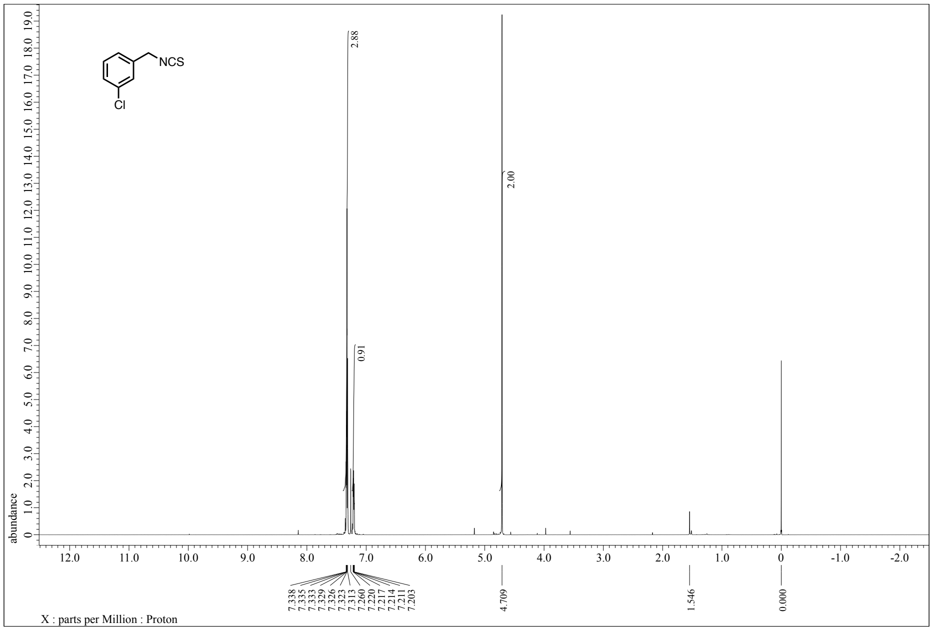

Supplementary Fig. 27: ^{1}H NMR (500 MHz) spectrum of **18**.

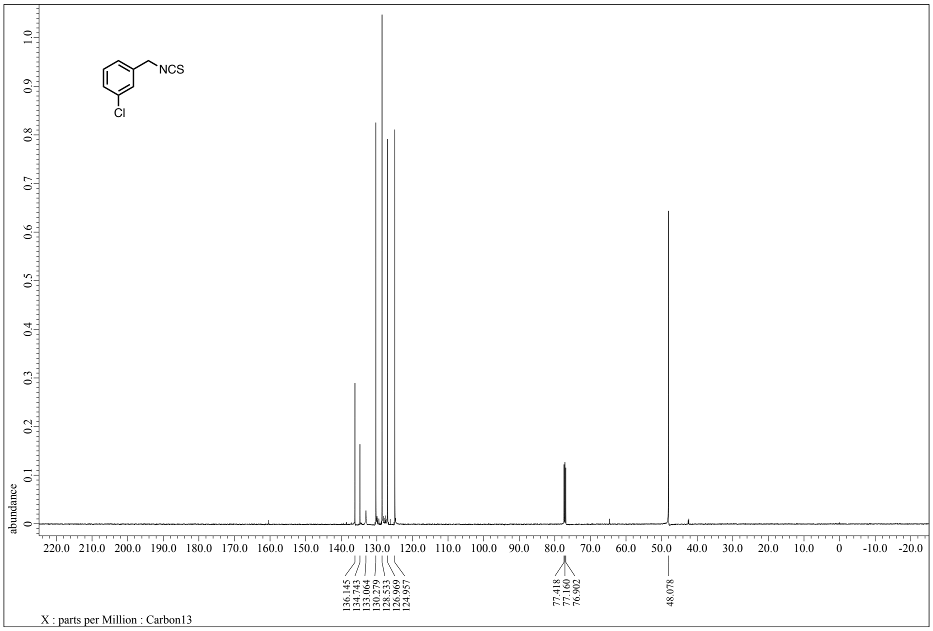

Supplementary Fig. 28: ^{13}C NMR (126 MHz) spectrum of **18**.

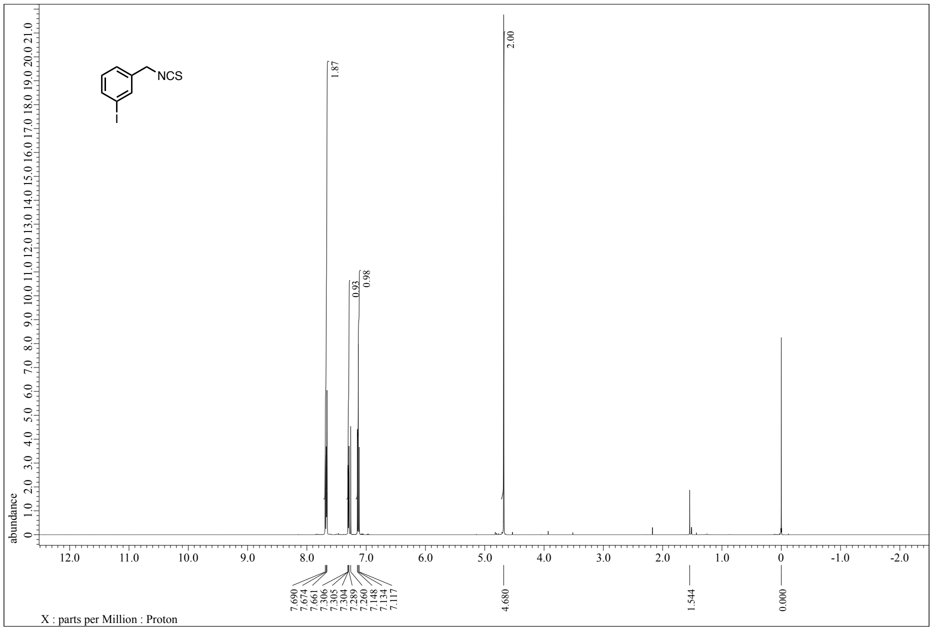

Supplementary Fig. 29: ^{1}H NMR (500 MHz) spectrum of **19**.

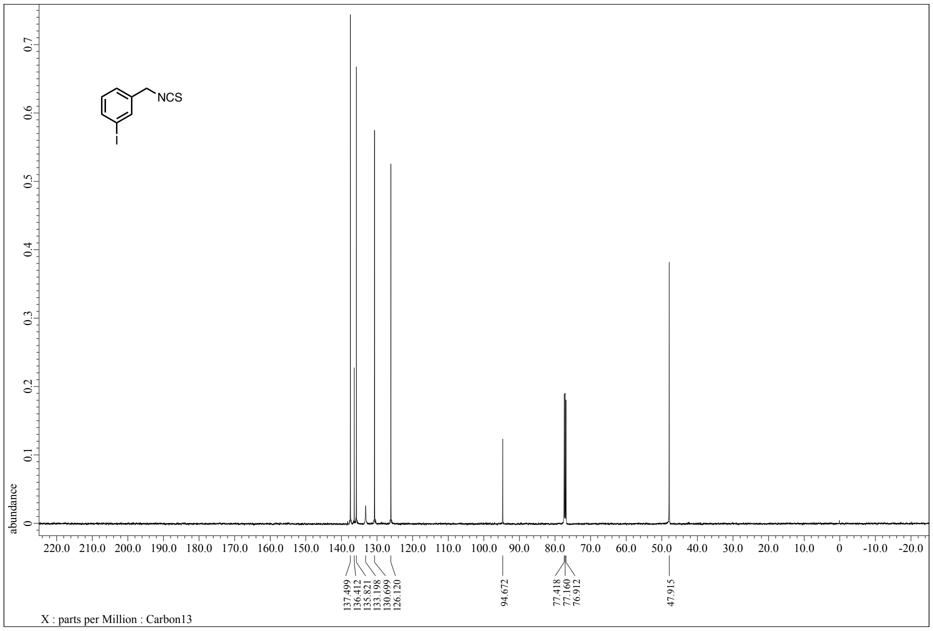

Supplementary Fig. 30: ^{13}C NMR (126 MHz) spectrum of **19**.

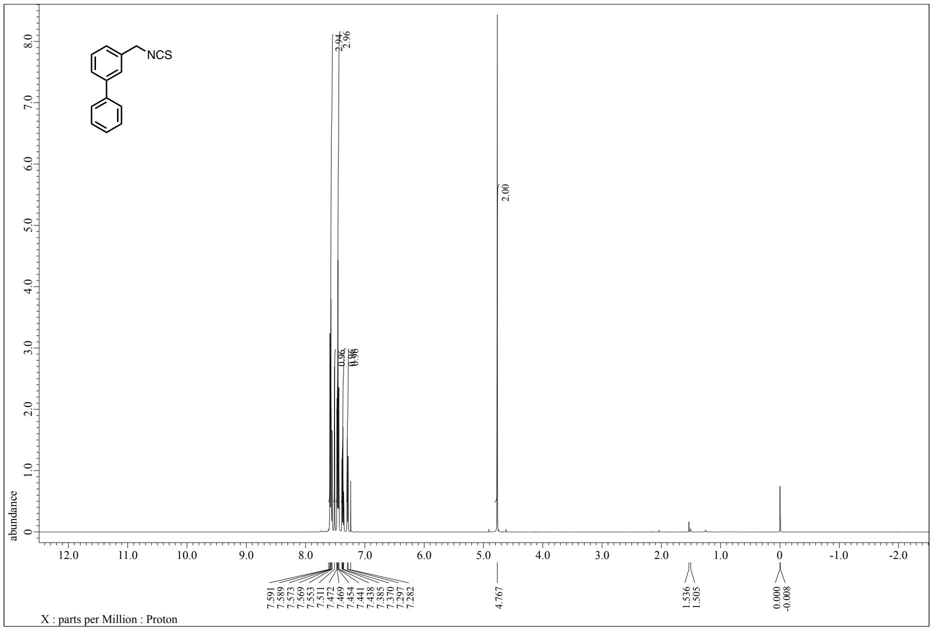

Supplementary Fig. 31: ^{1}H NMR (500 MHz) spectrum of **20**.

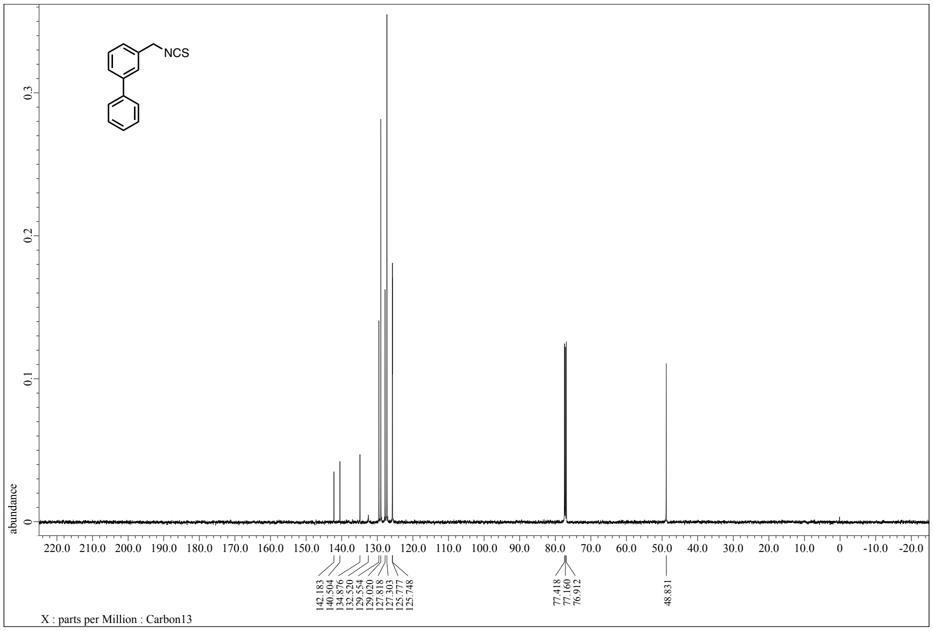

Supplementary Fig. 32: ^{13}C NMR (126 MHz) spectrum of **20**.

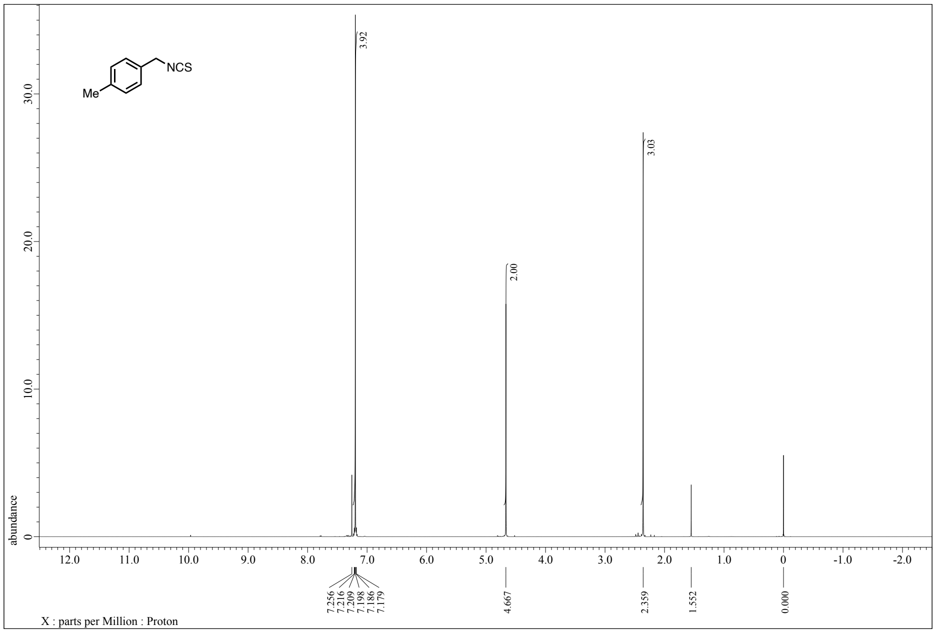

Supplementary Fig. 33: ^{1}H NMR (500 MHz) spectrum of **21**.

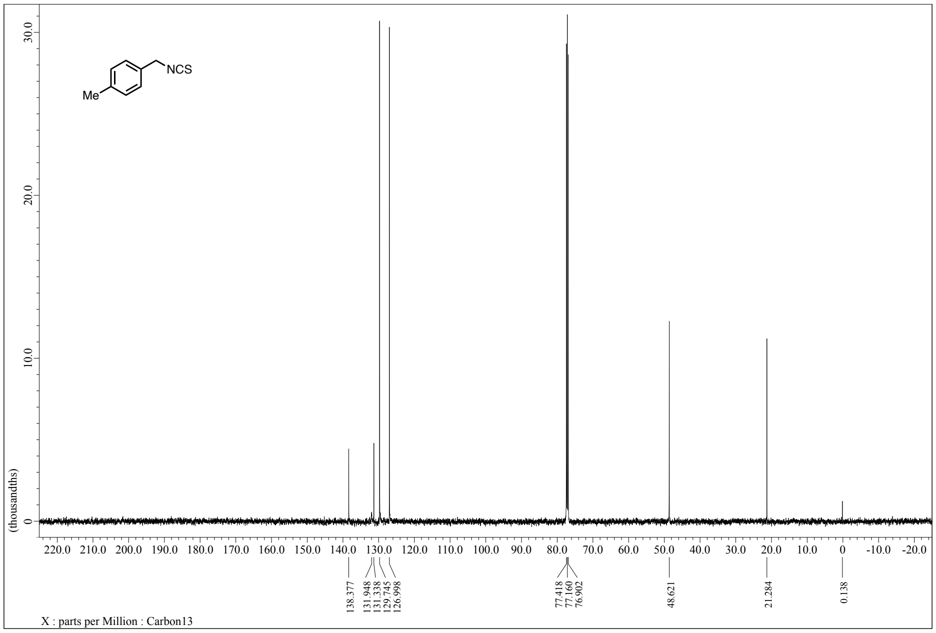

Supplementary Fig. 34: ^{13}C NMR (126 MHz) spectrum of **21**.

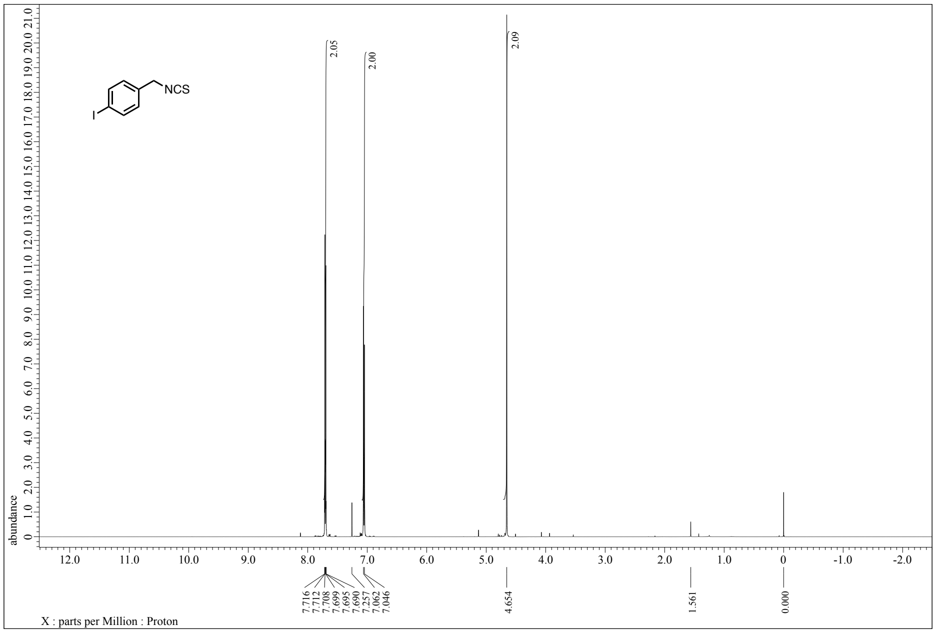

Supplementary Fig. 35: ^{1}H NMR (500 MHz) spectrum of **23**.

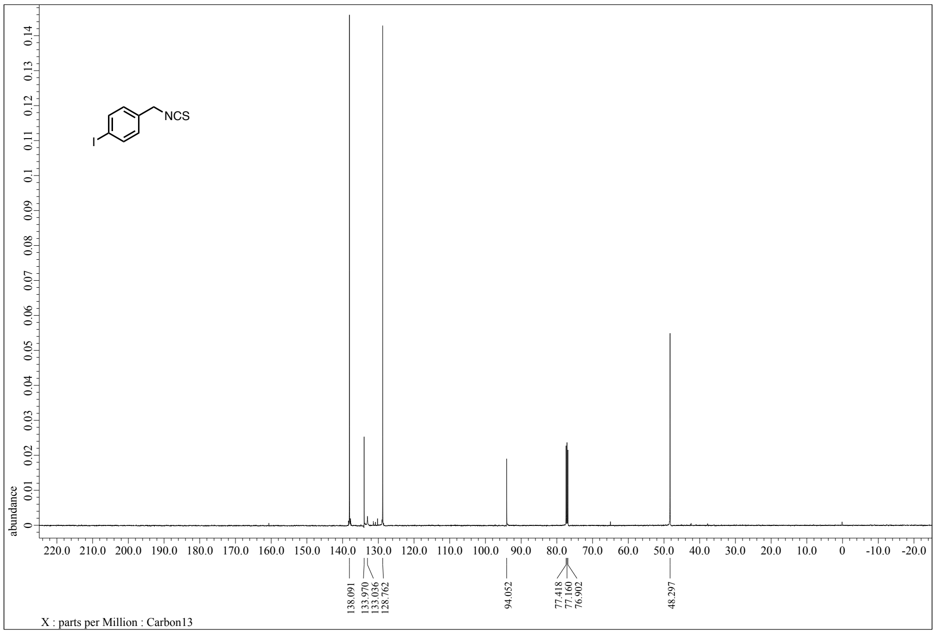

Supplementary Fig. 36: ^{13}C NMR (126 MHz) spectrum of **23**.

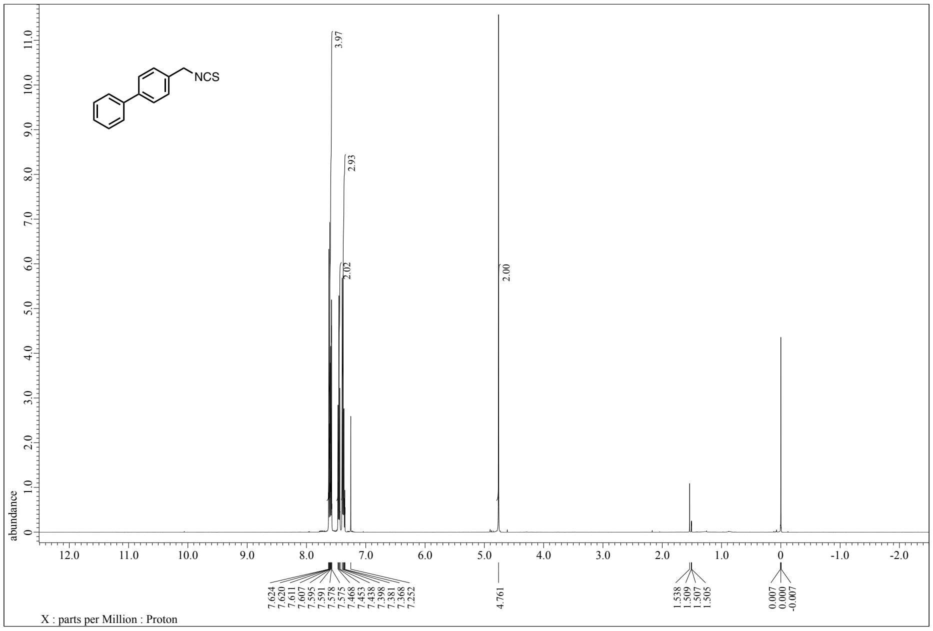

Supplementary Fig. 37: ^{1}H NMR (500 MHz) spectrum of **24**.

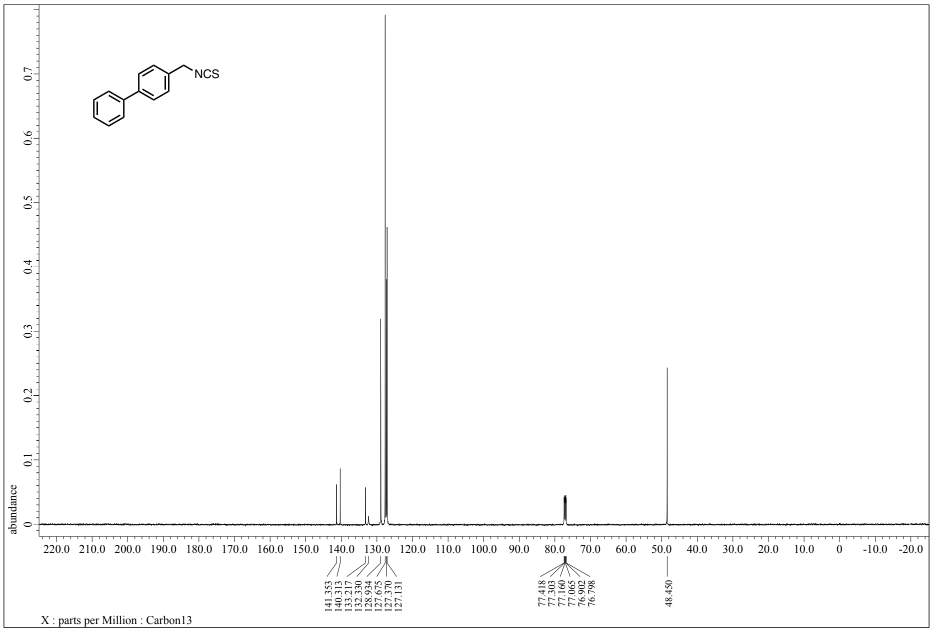

Supplementary Fig. 38: ^{13}C NMR (126 MHz) spectrum of **24**.

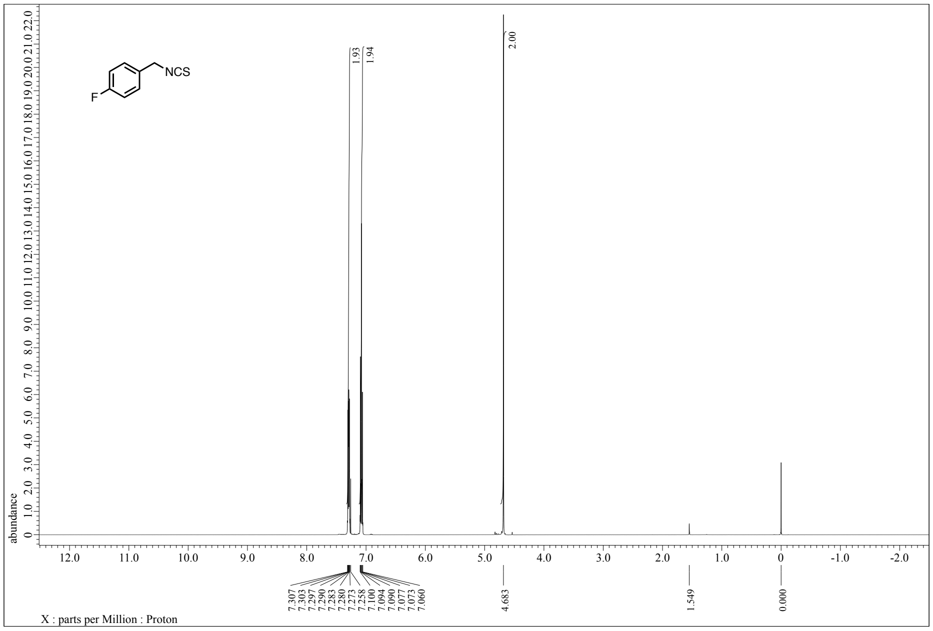

Supplementary Fig. 39: ^{1}H NMR (500 MHz) spectrum of **25**.

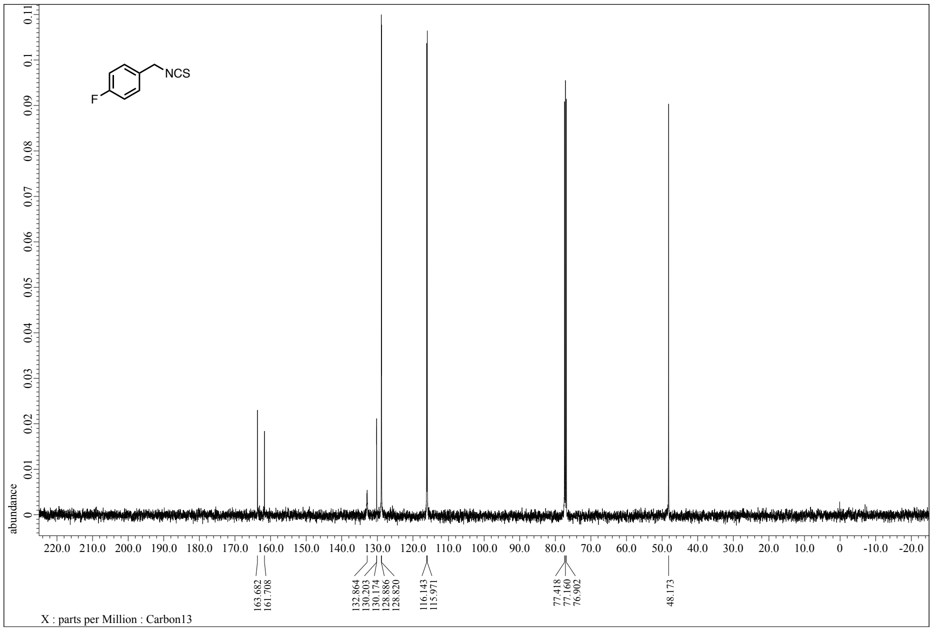

Supplementary Fig. 40: ^{13}C NMR (126 MHz) spectrum of **25**.

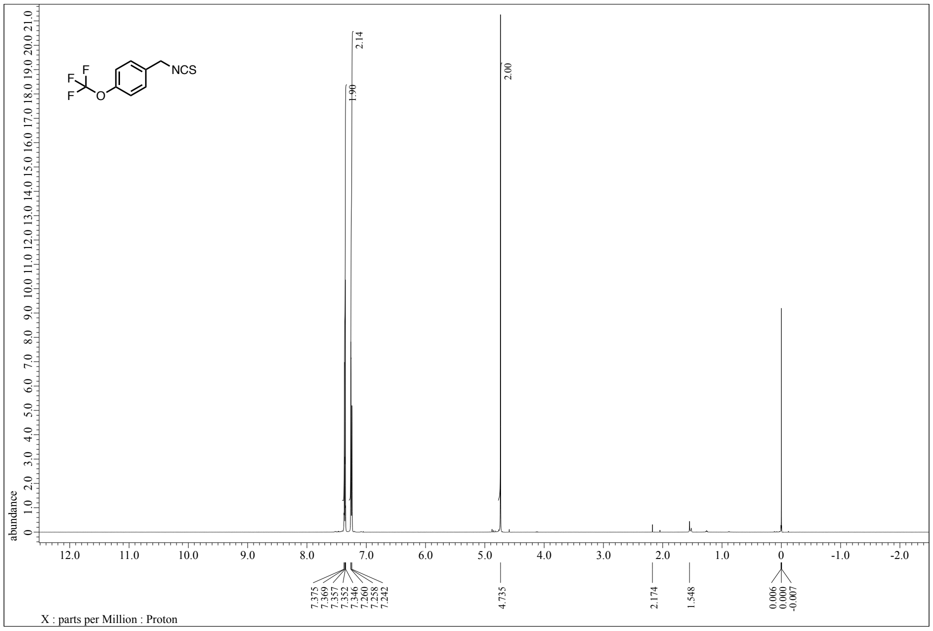

Supplementary Fig. 41: ^{1}H NMR (500 MHz) spectrum of **26**.

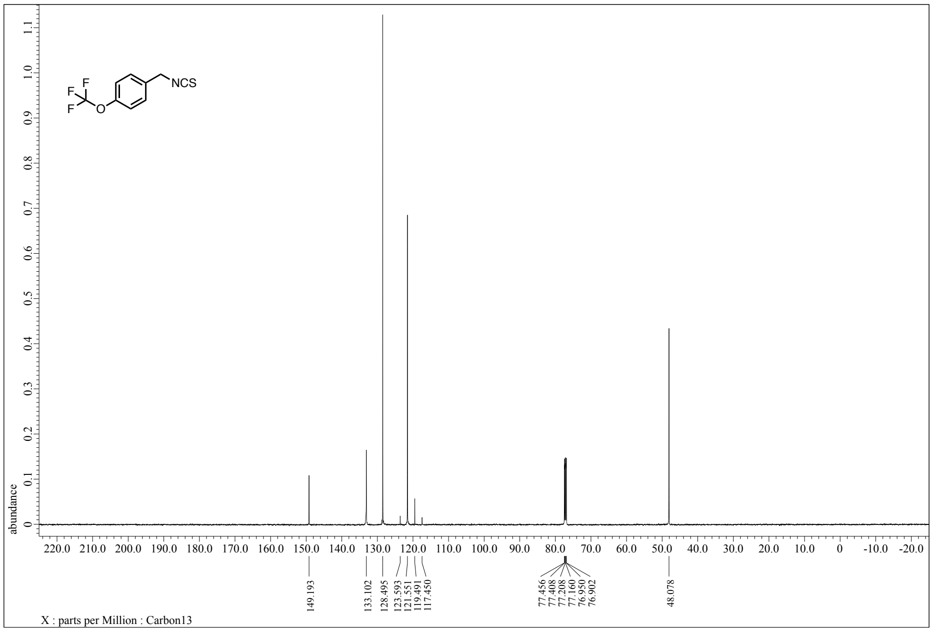

Supplementary Fig. 42: ^{13}C NMR (126 MHz) spectrum of **26**.

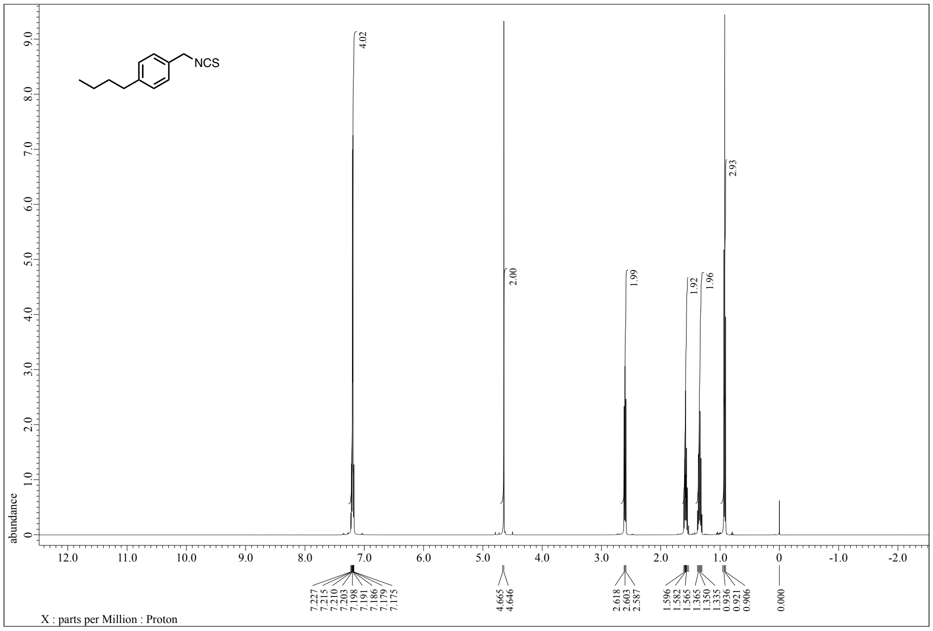

Supplementary Fig. 43: ^{1}H NMR (500 MHz) spectrum of **27**.

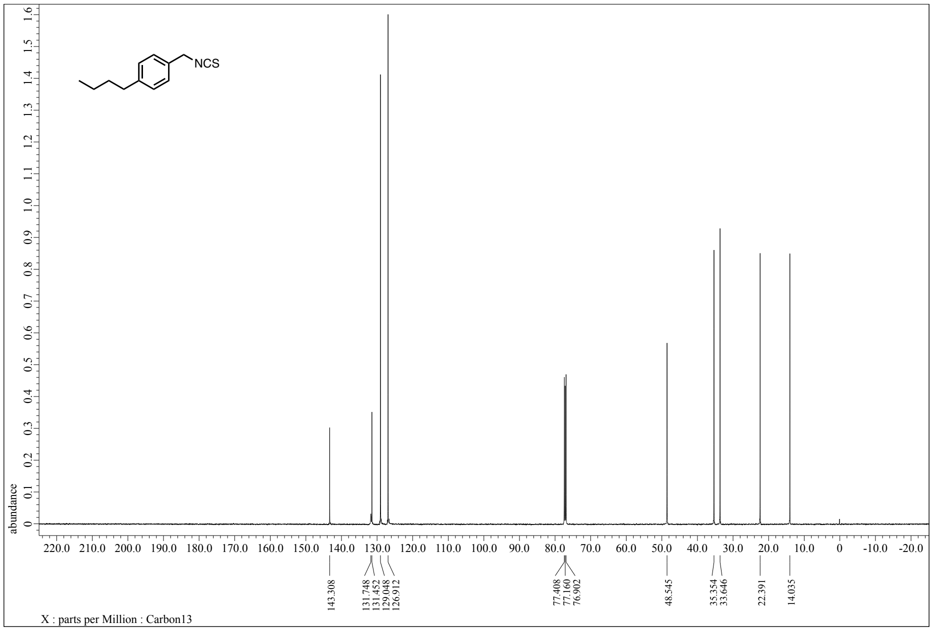

Supplementary Fig. 44: ^{13}C NMR (126 MHz) spectrum of **27**.

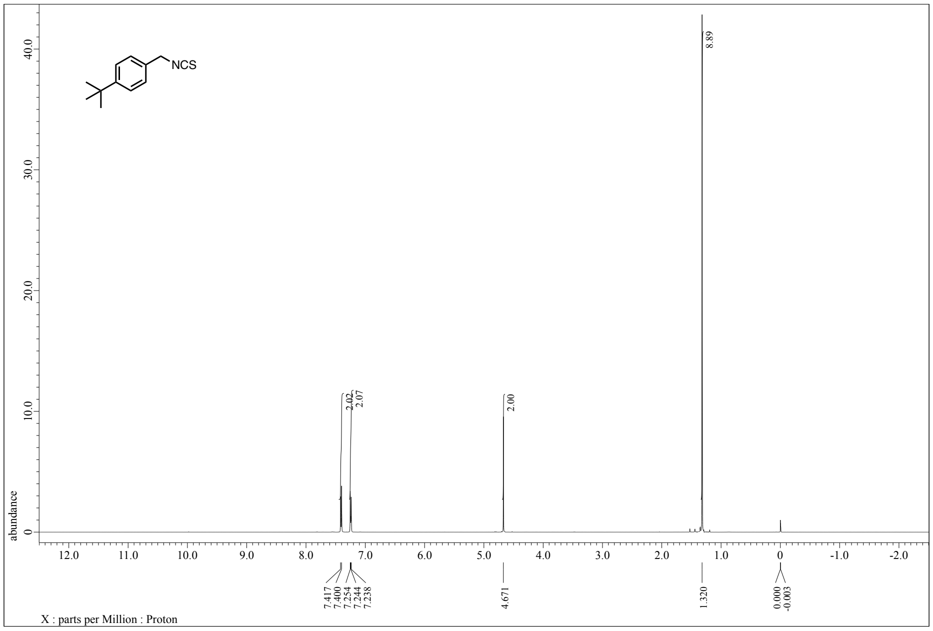

Supplementary Fig. 45: ^{1}H NMR (500 MHz) spectrum of **28**.

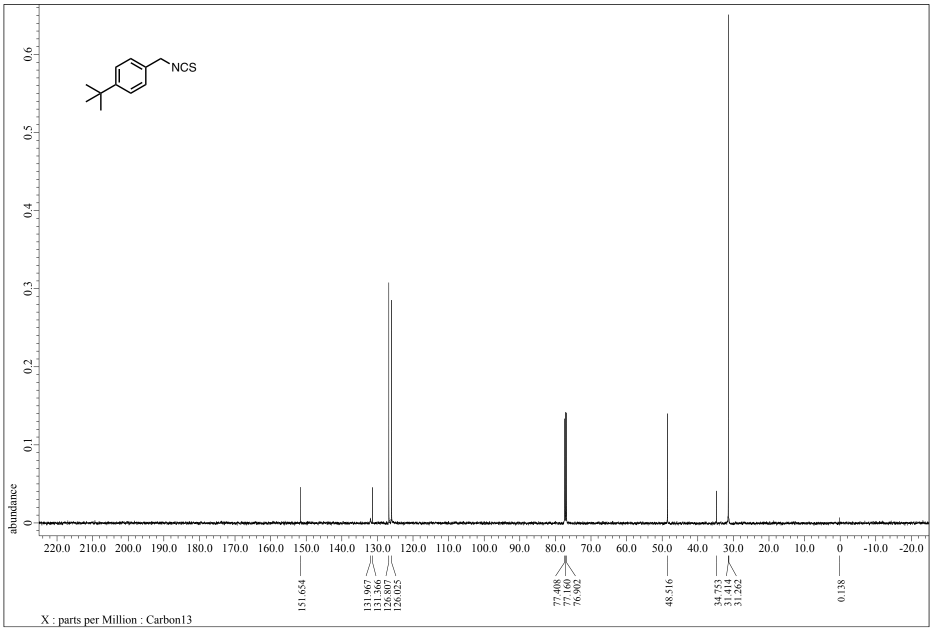

Supplementary Fig. 46: ^{13}C NMR (126 MHz) spectrum of **28**.

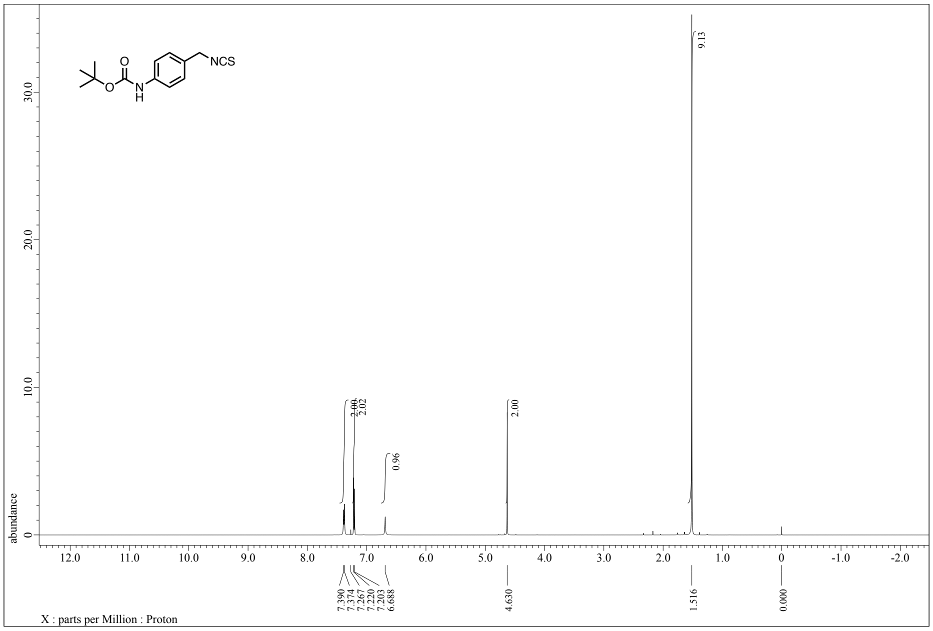

Supplementary Fig. 47: ^{1}H NMR (500 MHz) spectrum of **29**.

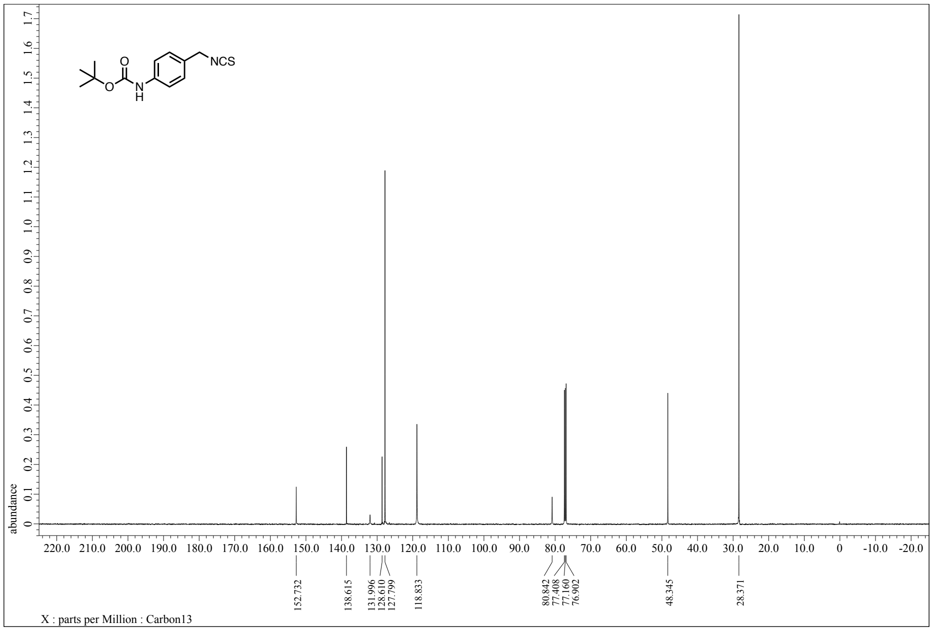

Supplementary Fig. 48: ^{13}C NMR (126 MHz) spectrum of **29**.

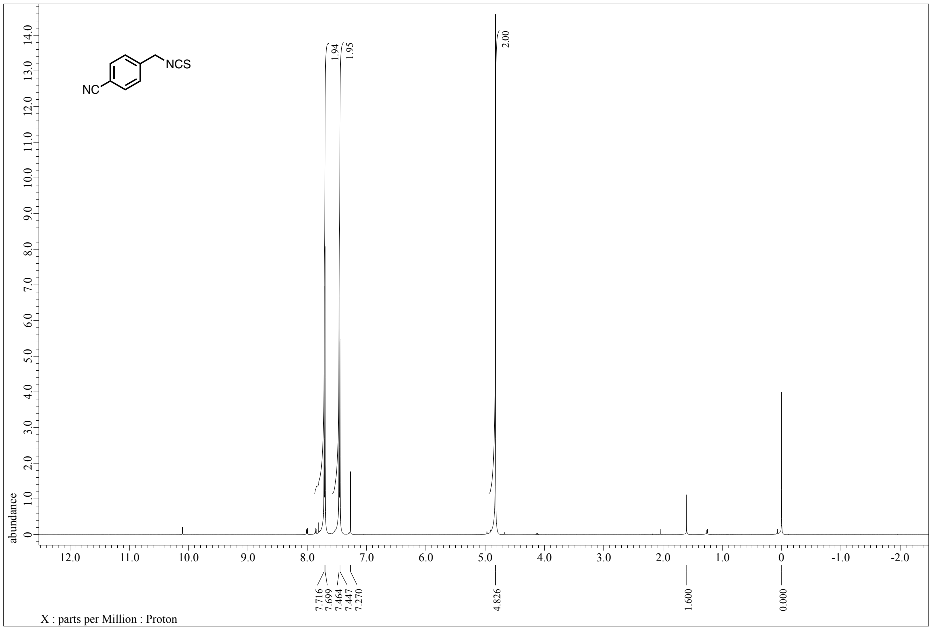

Supplementary Fig. 49: ^{1}H NMR (500 MHz) spectrum of **30**.

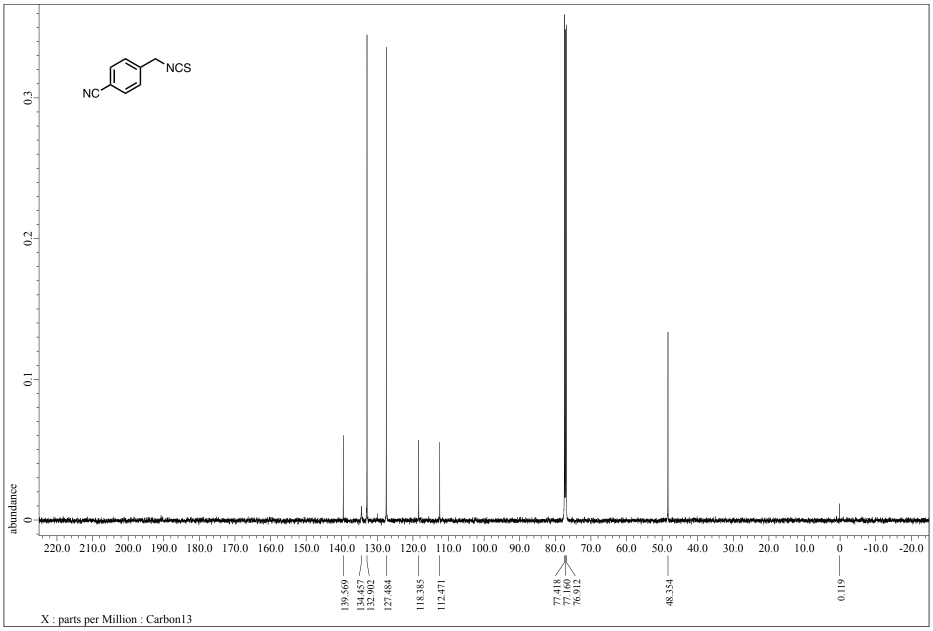

Supplementary Fig. 50: ^{13}C NMR (126 MHz) spectrum of **30**.

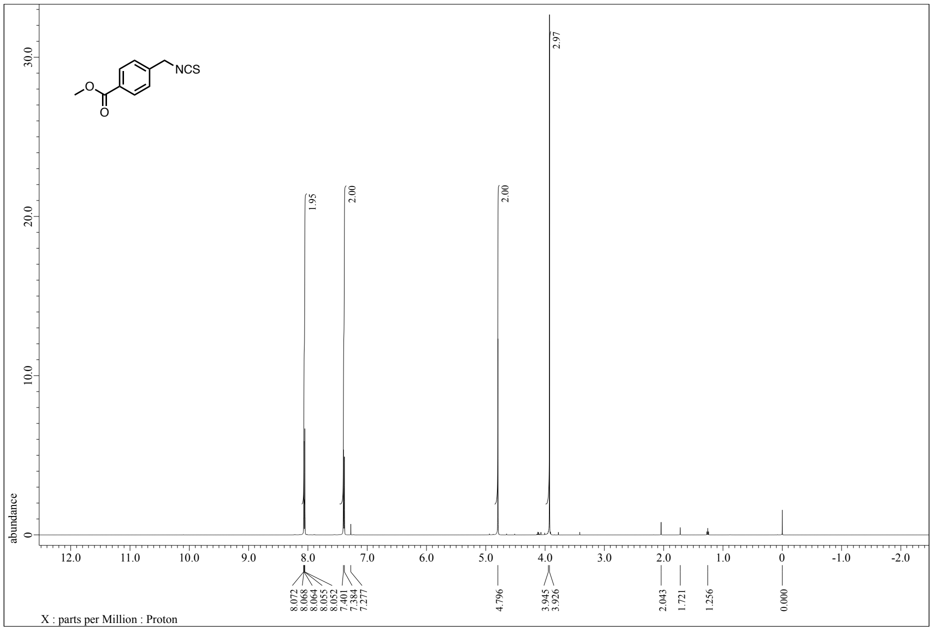

Supplementary Fig. 51: ^{1}H NMR (500 MHz) spectrum of **31**.

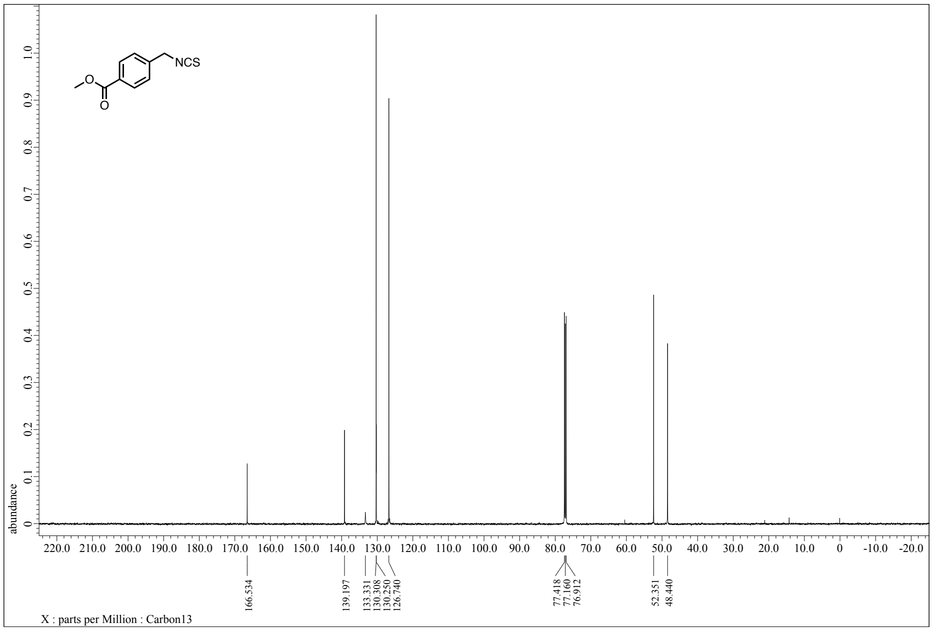

Supplementary Fig. 52: ^{13}C NMR (126 MHz) spectrum of **31**.

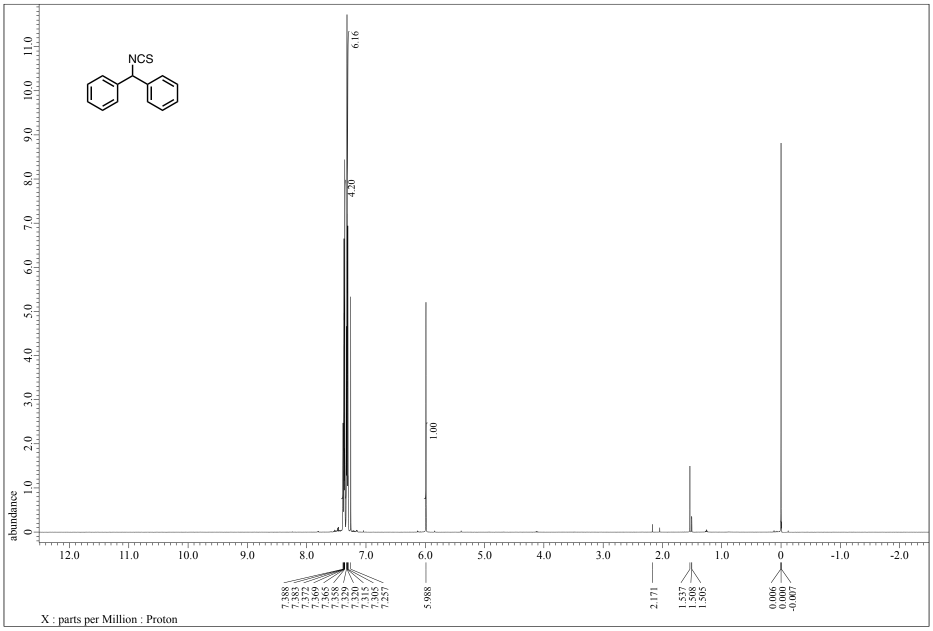

Supplementary Fig. 53: ^{1}H NMR (500 MHz) spectrum of **33**.

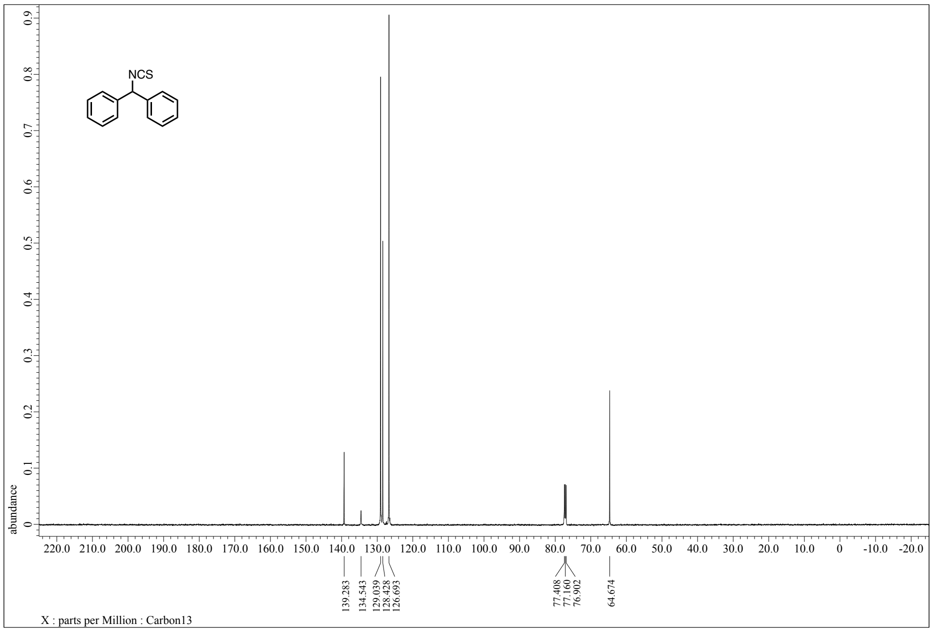

Supplementary Fig. 54: ^{13}C NMR (126 MHz) spectrum of **33**.

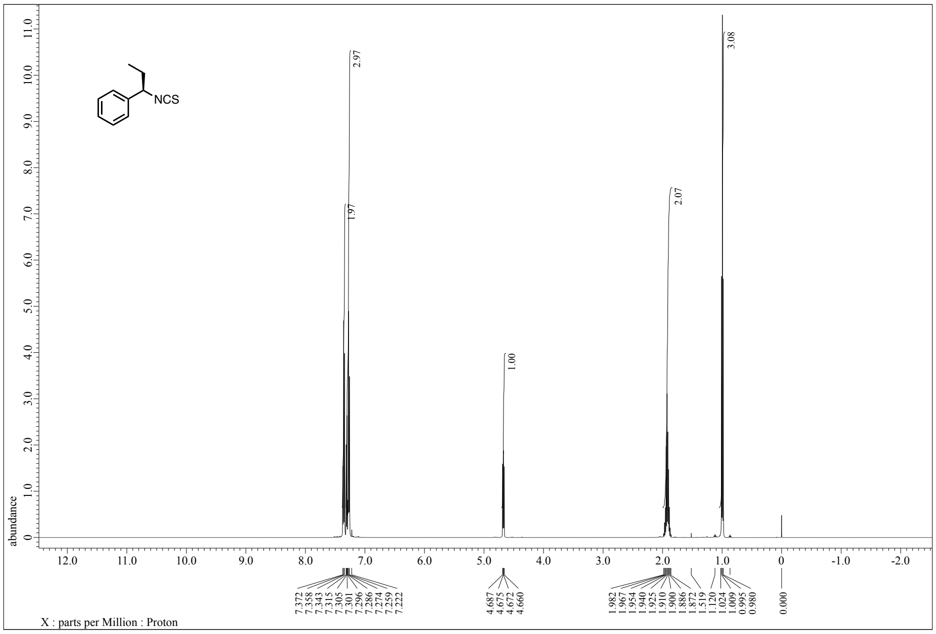

Supplementary Fig. 55: ^{1}H NMR (500 MHz) spectrum of **34**.

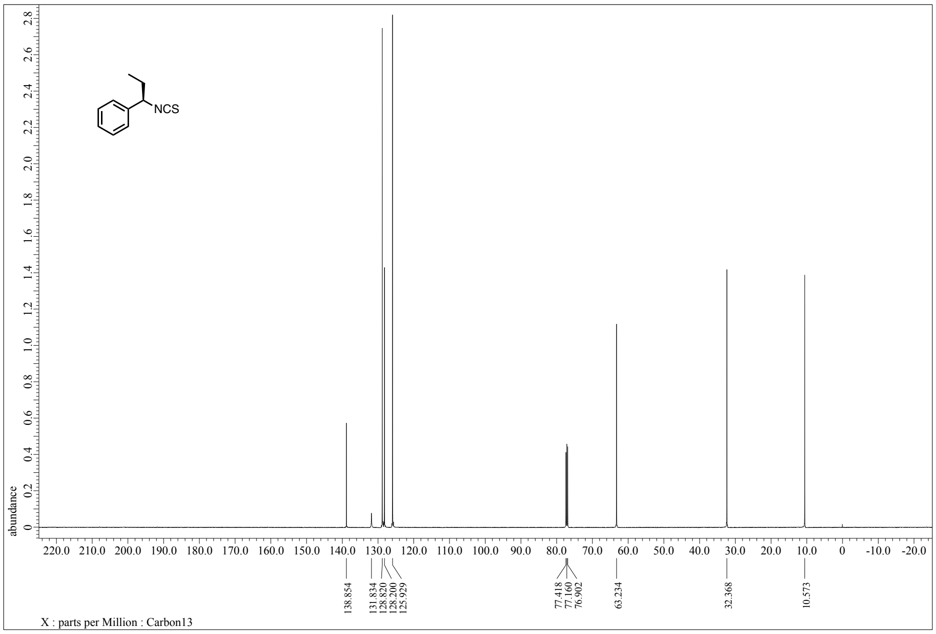

Supplementary Fig. 56: ^{13}C NMR (126 MHz) spectrum of **34**.

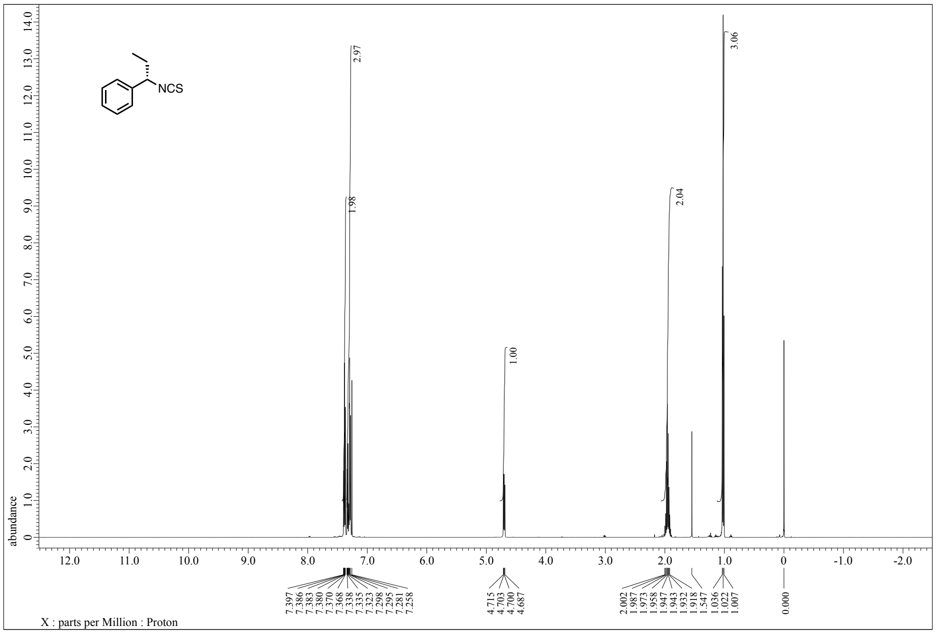

Supplementary Fig. 57: ^{1}H NMR (500 MHz) spectrum of **35**.

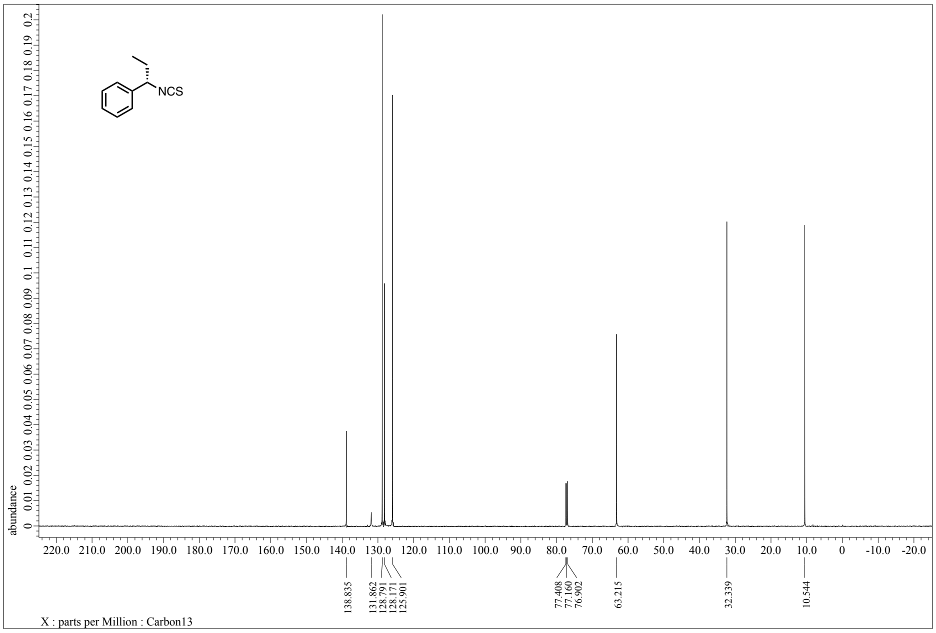

Supplementary Fig. 58: ^{13}C NMR (126 MHz) spectrum of **35**.

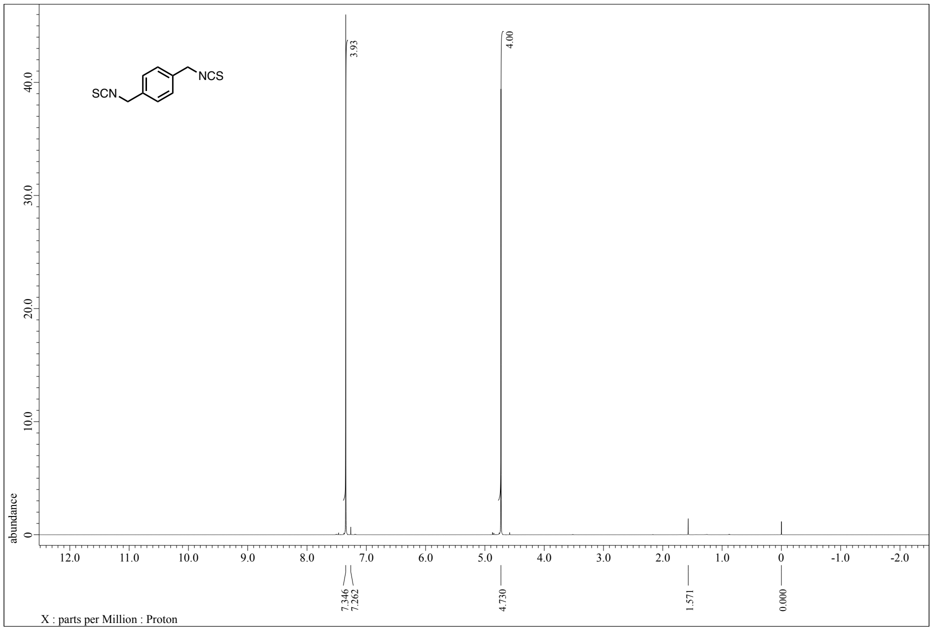

Supplementary Fig. 59: ^{1}H NMR (500 MHz) spectrum of **37**.

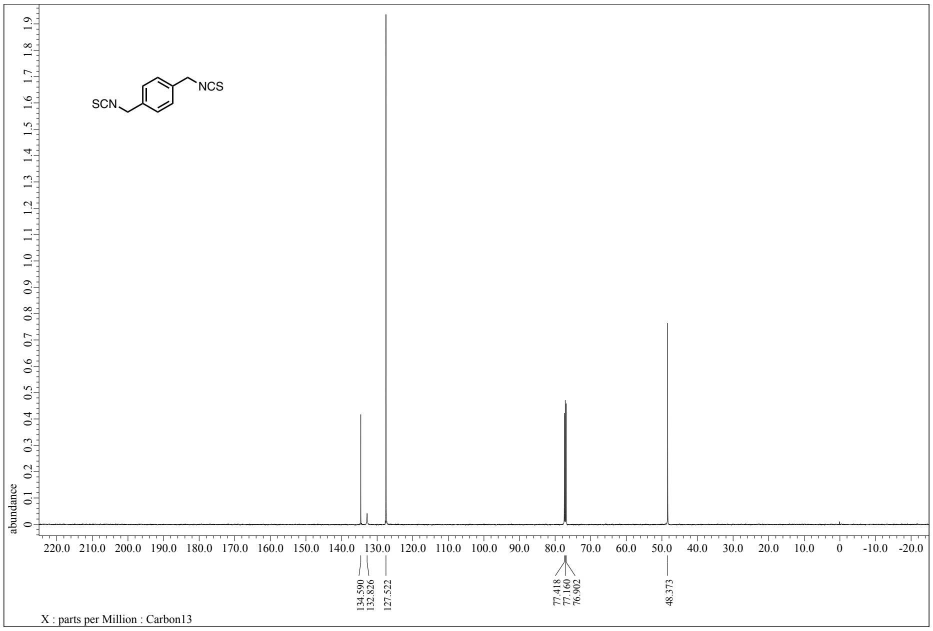

Supplementary Fig. 60: ^{13}C NMR (126 MHz) spectrum of **37**.

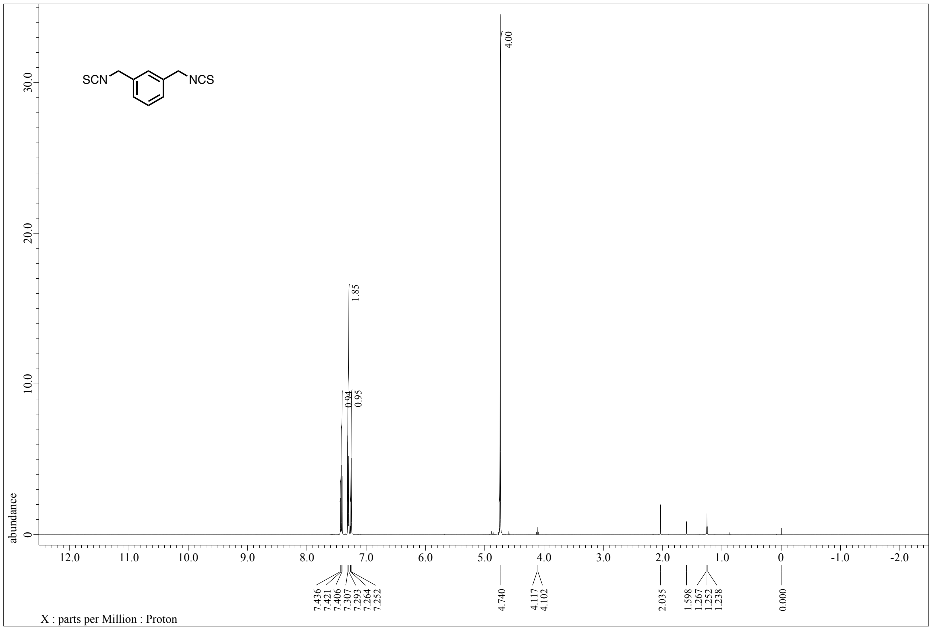

Supplementary Fig. 61: ^{1}H NMR (500 MHz) spectrum of **38**.

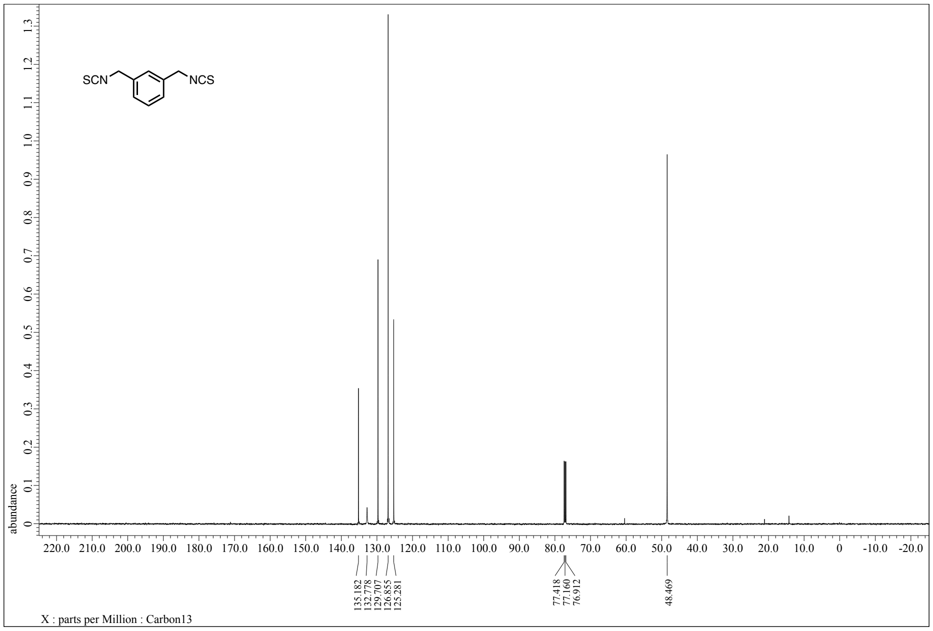

Supplementary Fig. 62: ^{13}C NMR (126 MHz) spectrum of **38**.

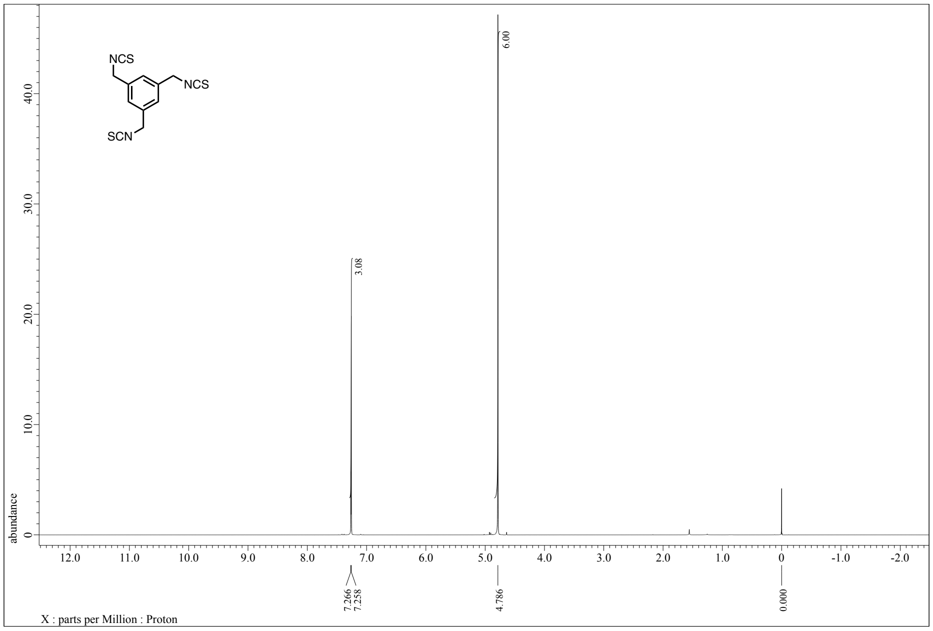

Supplementary Fig. 63: ^{1}H NMR (500 MHz) spectrum of **39**.

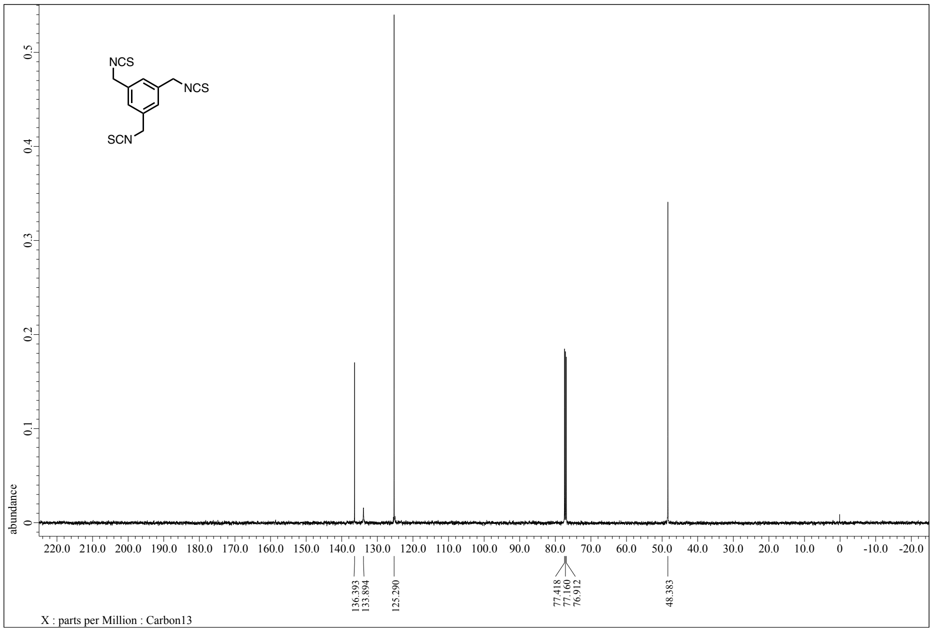

Supplementary Fig. 64: ^{13}C NMR (126 MHz) spectrum of **39**.

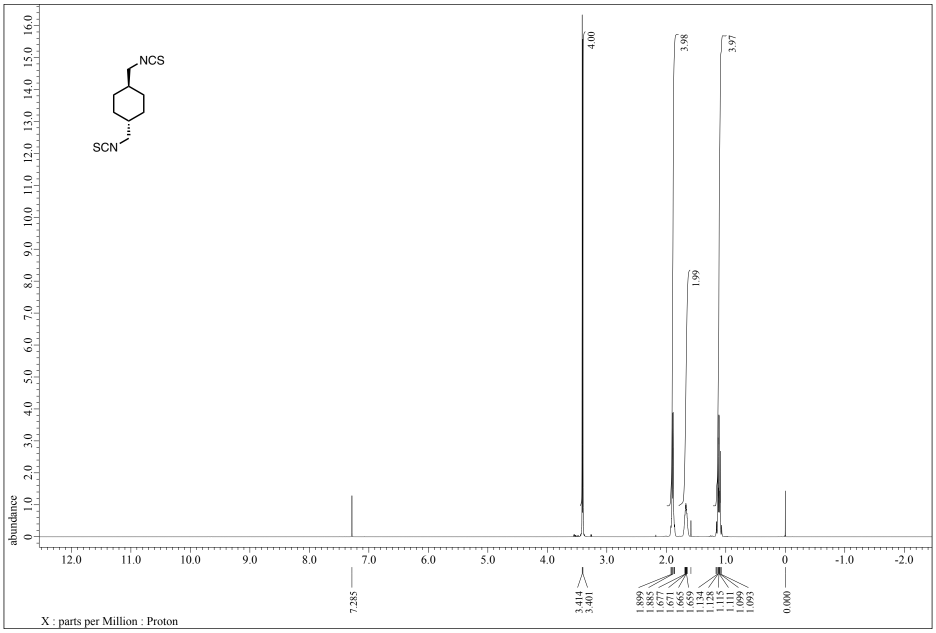

Supplementary Fig. 65: ^{1}H NMR (500 MHz) spectrum of **40**.

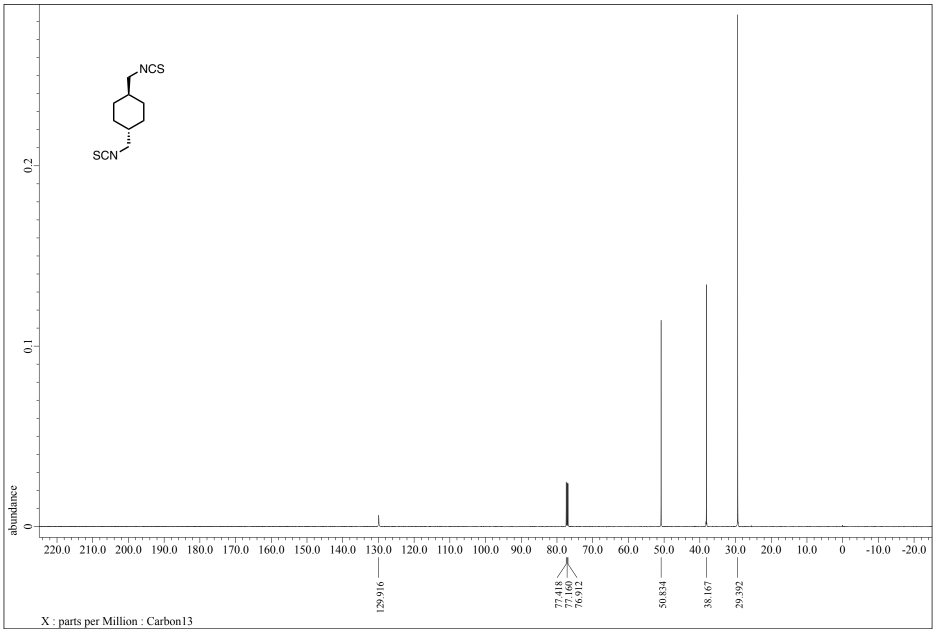

Supplementary Fig. 66: ^{13}C NMR (126 MHz) spectrum of **40**.

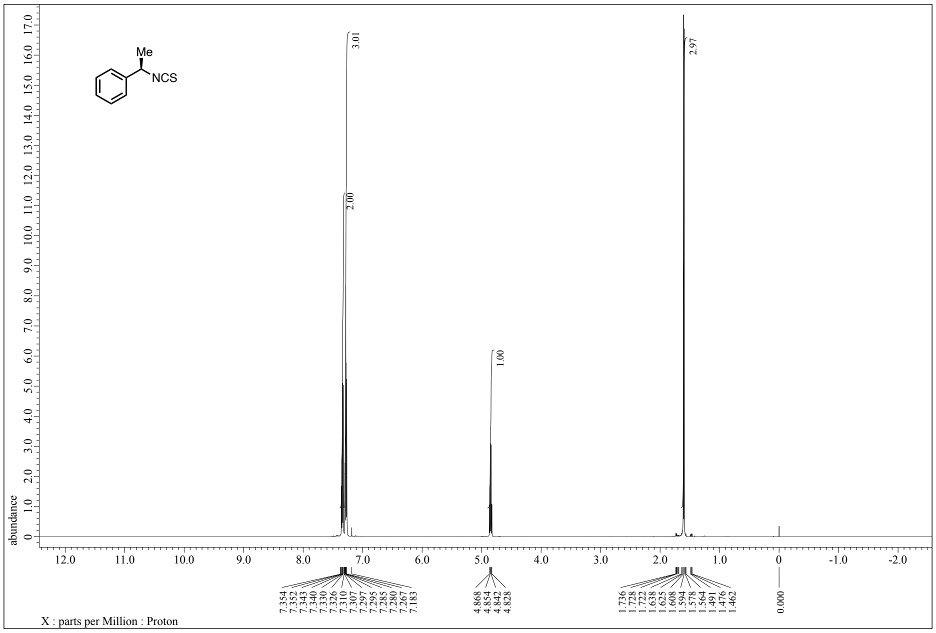

Supplementary Fig. 67: ^{1}H NMR (500 MHz) spectrum of **S1**.

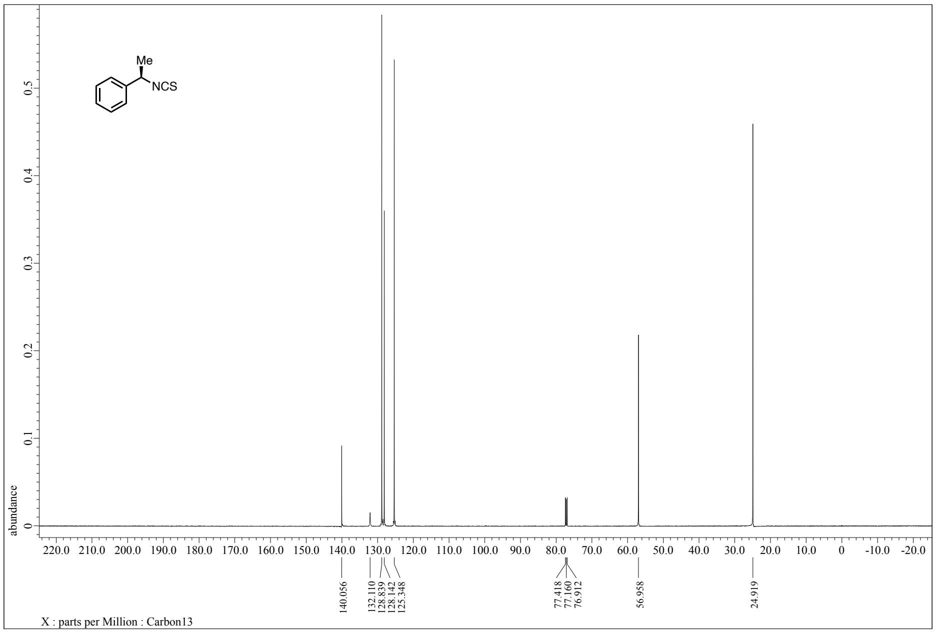

Supplementary Fig. 68: ^{13}C NMR (126 MHz) spectrum of **S1**.

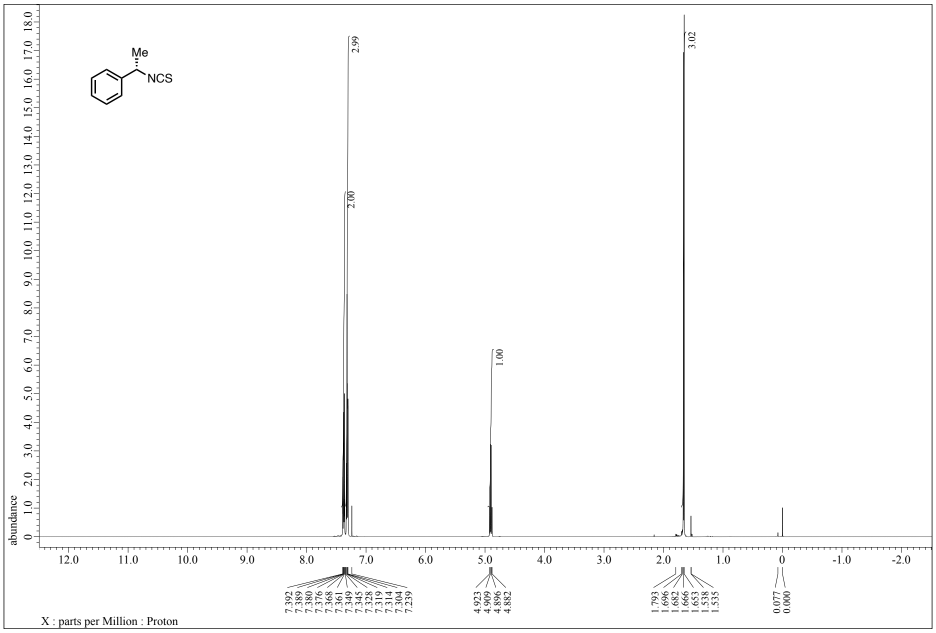

Supplementary Fig. 69: ^{1}H NMR (500 MHz) spectrum of **S2**.

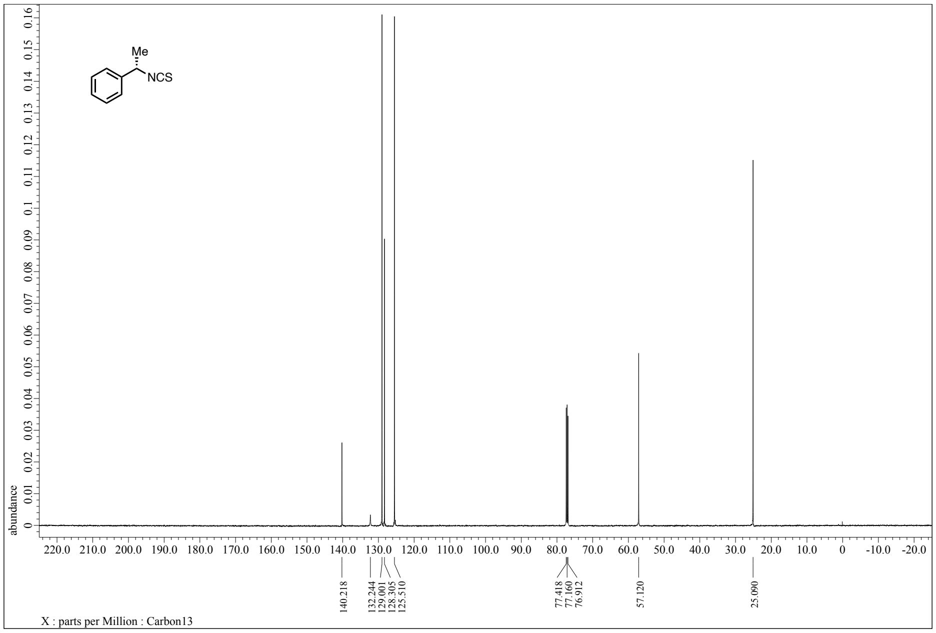

Supplementary Fig. 70: ^{13}C NMR (126 MHz) spectrum of **S2**.

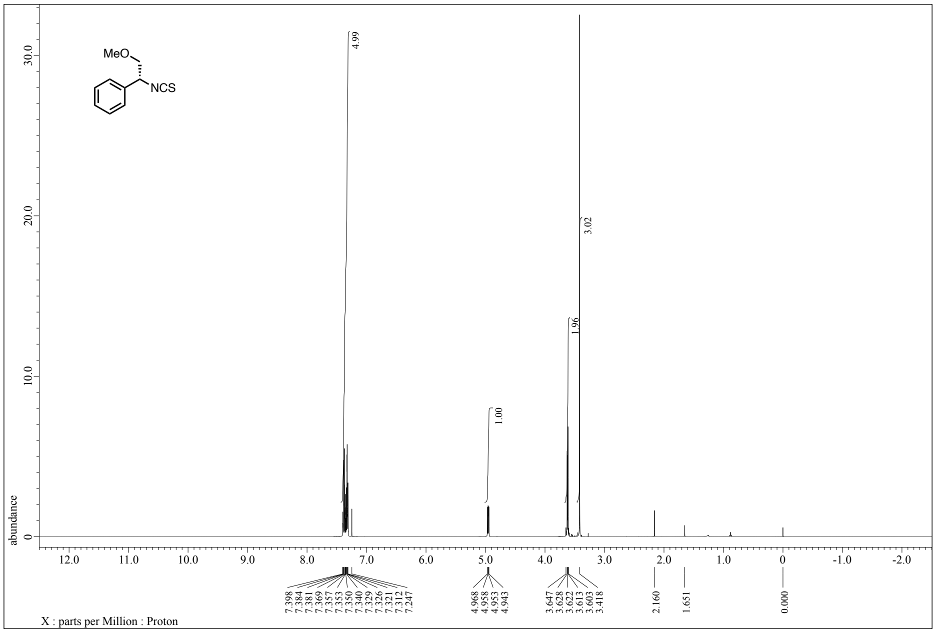

Supplementary Fig. 71: ^{1}H NMR (500 MHz) spectrum of **S3**.

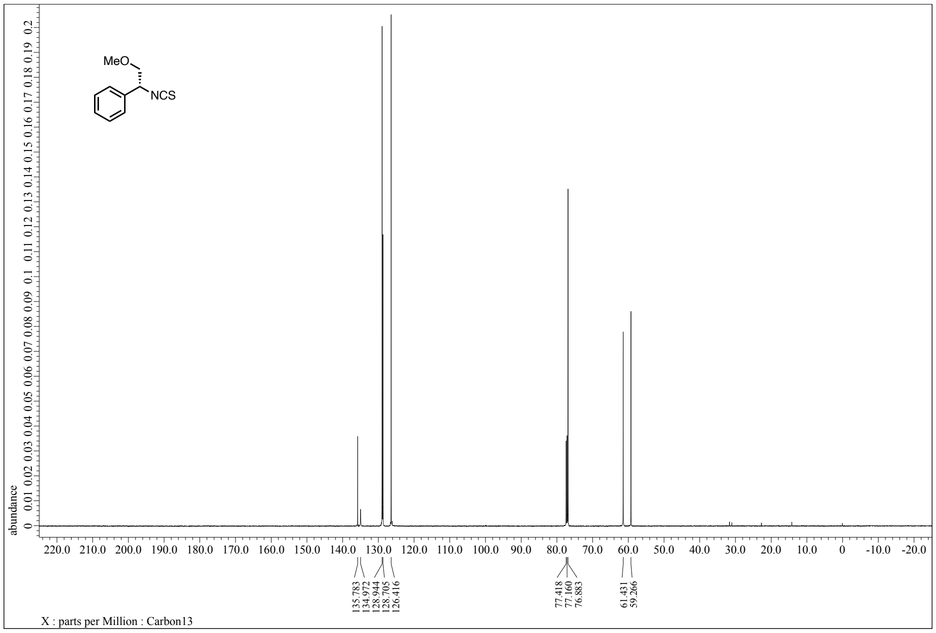

Supplementary Fig. 72: ^{13}C NMR (126 MHz) spectrum of **S3**.

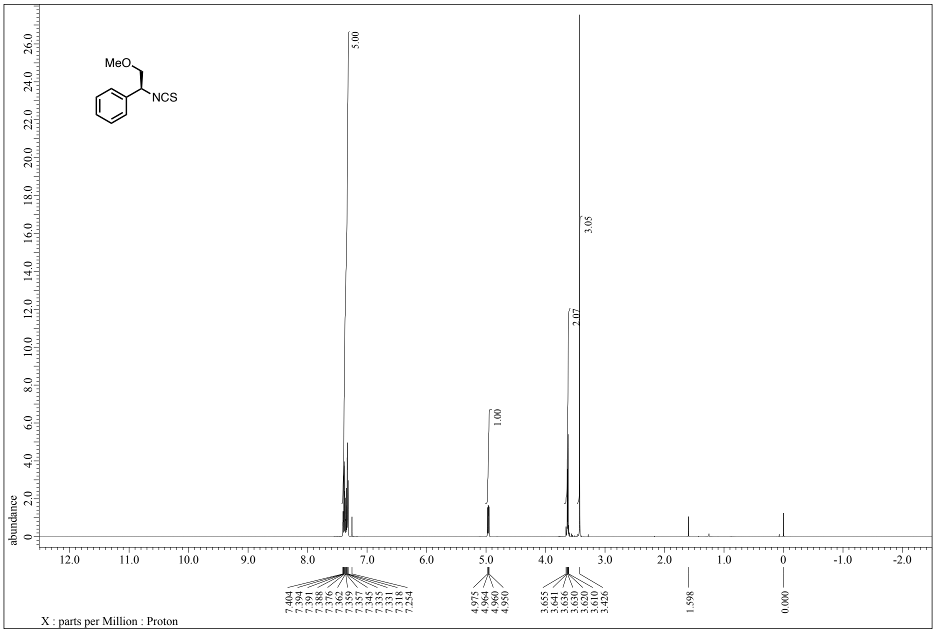

Supplementary Fig. 73: ^{1}H NMR (500 MHz) spectrum of **S4**.

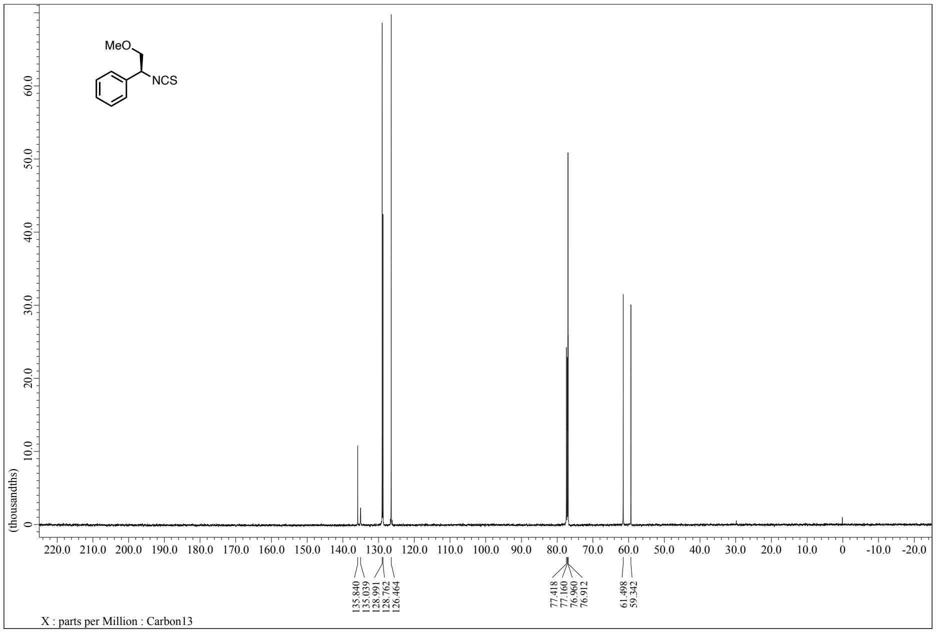

Supplementary Fig. 74: ^{13}C NMR (126 MHz) spectrum of **S4**.

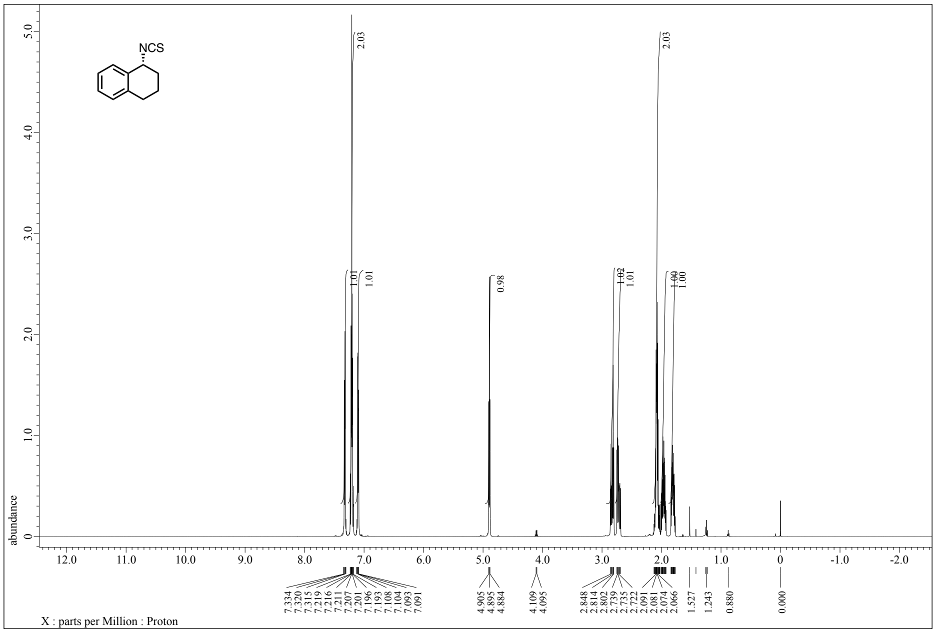

Supplementary Fig. 75: ^{1}H NMR (500 MHz) spectrum of **S5**.

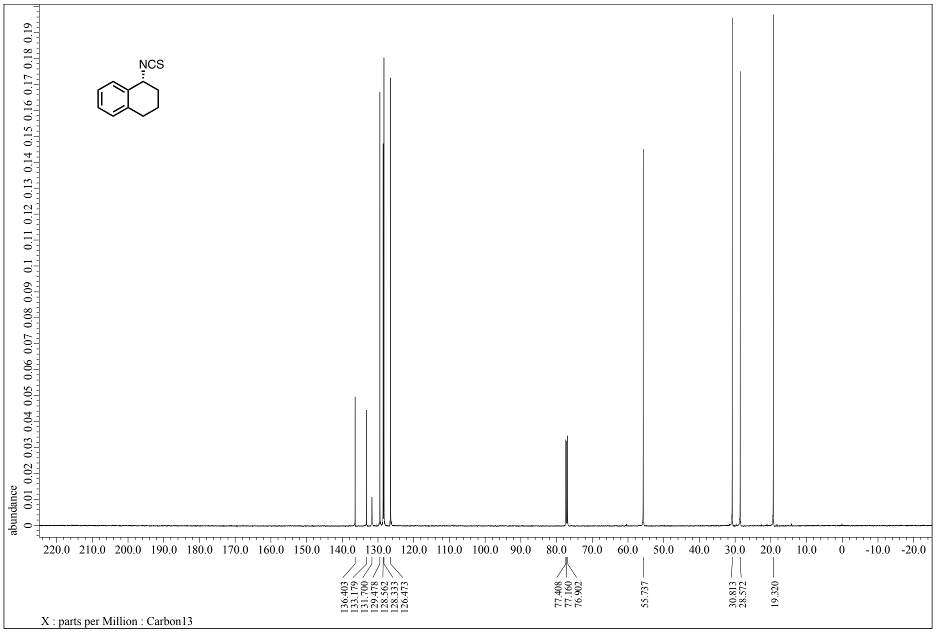

Supplementary Fig. 76: ^{13}C NMR (126 MHz) spectrum of **S5**.

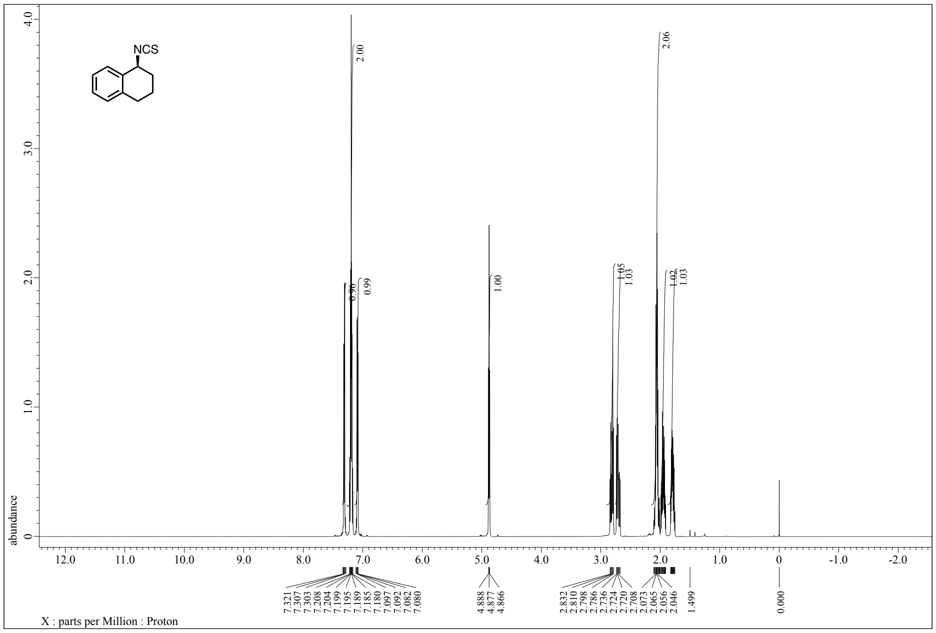

Supplementary Fig. 77: ^{1}H NMR (500 MHz) spectrum of **S6**.

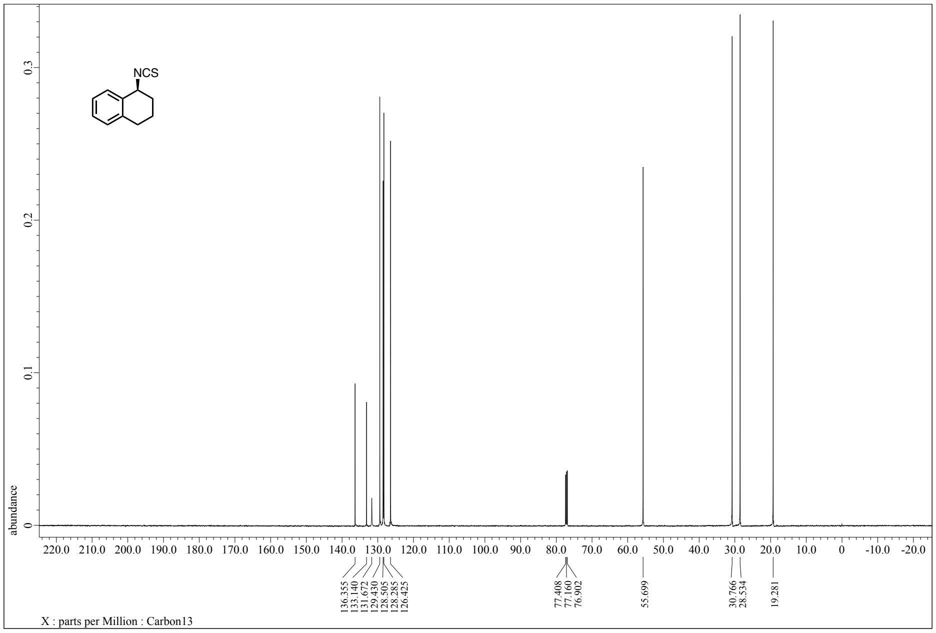

Supplementary Fig. 78: ^{13}C NMR (126 MHz) spectrum of **S6**.

Supplementary References

1. Chen, L. *et al.* TCS1, a Microtubule-Binding Protein, Interacts with KCBP/ZWICHEL to Regulate Trichome Cell Shape in Arabidopsis thaliana. *PLOS Genetics* **12**, e1006266 (2016).
2. Fu, Z., Yuan, W., Chen, N., Yang, Z. & Xu, J. Na2S2O8-mediated efficient synthesis of isothiocyanates from primary amines in water. *Green Chem.* **20**, 4484–4491 (2018).
3. Spencer, E. S. *et al.* Multiple binding modes of isothiocyanates that inhibit macrophage migration inhibitory factor. *Eur. J. Med. Chem.* **93**, 501–510 (2015).
4. Anderson, R. H. *et al.* Differentiating Antiproliferative and Chemopreventive Modes of Activity for Electron-Deficient Aryl Isothiocyanates against Human MCF-7 Cells. *ChemMedChem* **13**, 1695–1710 (2018).
5. Mays, J. R., Weller Roska, R. L., Sarfaraz, S., Mukhtar, H. & Rajski, S. R. Identification, synthesis, and enzymology of non-natural glucosinolate chemopreventive candidates. *Chembiochem* **9**, 729–747 (2008).
6. Kim, T. *et al.* The synthesis of sulforaphane analogues and their protection effect against cisplatin induced cytotoxicity in kidney cells. *Bioorganic & Medicinal Chemistry Letters* **25**, 62–66 (2015).
7. García-Reyes, B. *et al.* Discovery of Inhibitor of Wnt Production 2 (IWP-2) and Related Compounds As Selective ATP-Competitive Inhibitors of Casein Kinase 1 (CK1) δ/ε. *J. Med. Chem.* **61**, 4087–4102 (2018).
8. More, K. N. *et al.* Effect of TRPV1 Antagonist SC0030, a Potent Painkiller, on RANKL-mediated Osteoclast Differentiation Involved in Bone Resorption. *Bull. Korean Chem. Soc.* **41**, 488–491 (2020).
9. Luo, B. *et al.* New Mild and Simple Approach to Isothiocyanates: A Class of Potent Anticancer Agents. *Molecules* **22**, 773 (2017).
