# Supplementary material for: Identification and improvement of isothiocyanate-based inhibitors on stomatal opening to act as drought tolerance-conferring agrochemicals
Source: Nat Commun. 2023 May 15;14:2665. doi: 10.1038/s41467-023-38102-7 (PMC10185662; doi:10.1038/s41467-023-38102-7)
Supplement: Supplementary file 3 — Description of Additional Supplementary Files [file 41467_2023_38102_MOESM3_ESM.pdf]

Description of Additional Supplementary Files:

Supplementary Data 1. List of Differentially Expressed Genes and enriched GOs analyzed for RNA-seq experiment in Figure 5.

Supplementary Movie 1. Real-time observation of the effect of BITC and m-bis-BITC on Chrysanthemum leaf wilting. Experimental conditions are the same as in Figure 5C. The movie was recorded at 60x speed for 1.5 h real-time.
