# Supplementary material for: Identification and improvement of isothiocyanate-based inhibitors on stomatal opening to act as drought tolerance-conferring agrochemicals
Source: Nat Commun. 2023 May 15;14:2665. doi: 10.1038/s41467-023-38102-7 (PMC10185662; doi:10.1038/s41467-023-38102-7)
Supplement: Supplementary file 6 — Reporting Summary [file 41467_2023_38102_MOESM6_ESM.pdf]

nature portfolio

Corresponding author(s): Toshinori Kinoshita, Kei Murakami

Last updated by author(s): 2023-Apr-2

Reporting Summary

Nature Portfolio wishes to improve the reproducibility of the work that we publish. This form provides structure for consistency and transparency in reporting. For further information on Nature Portfolio policies, see our Editorial Policies and the Editorial Policy Checklist.

Statistics

For all statistical analyses, confirm that the following items are present in the figure legend, table legend, main text, or Methods section.

n/a	Confirmed	
☐	☒	The exact sample size (*n*) for each experimental group/condition, given as a discrete number and unit of measurement
☐	☒	A statement on whether measurements were taken from distinct samples or whether the same sample was measured repeatedly
☐	☒	The statistical test(s) used AND whether they are one- or two-sided<br>*Only common tests should be described solely by name; describe more complex techniques in the Methods section.*
☒	☐	A description of all covariates tested
☒	☐	A description of any assumptions or corrections, such as tests of normality and adjustment for multiple comparisons
☐	☒	A full description of the statistical parameters including central tendency (e.g. means) or other basic estimates (e.g. regression coefficient) AND variation (e.g. standard deviation) or associated estimates of uncertainty (e.g. confidence intervals)
☒	☐	For null hypothesis testing, the test statistic (e.g. *F*, *t*, *r*) with confidence intervals, effect sizes, degrees of freedom and *P* value noted<br>*Give P values as exact values whenever suitable.*
☒	☐	For Bayesian analysis, information on the choice of priors and Markov chain Monte Carlo settings
☒	☐	For hierarchical and complex designs, identification of the appropriate level for tests and full reporting of outcomes
☐	☒	Estimates of effect sizes (e.g. Cohen's *d*, Pearson's *r*), indicating how they were calculated

Our web collection on statistics for biologists contains articles on many of the points above.

Software and code

Policy information about availability of computer code

Data collection	Microsoft Excel for Mac v16.68 were used to analyze the data.
Data analysis	Microsoft Excel for Mac 2011 v14.7.7 was used for statistical analyses.<br>Prism 9 (v.9.3.0, GraphPad Software, LLC.) was used for sigmoid fitting analysis or to draw the graphs.<br>The EdgeR package on the web (Degust, https://degust.erc.monash.edu/) was used to for pairwise comparisons between RNA-seq samples data.<br>DeepStomata was used to automatically detect and measure stomatal apertures of Commelina benghalensis.<br>"Draw Venn Diagram" (https://bioinformatics.psb.ugent.be/webtools/Venn/) was used to draw Venn diagram.

For manuscripts utilizing custom algorithms or software that are central to the research but not yet described in published literature, software must be made available to editors and reviewers. We strongly encourage code deposition in a community repository (e.g. GitHub). See the Nature Portfolio guidelines for submitting code & software for further information.

Data

Policy information about availability of data

All manuscripts must include a data availability statement. This statement should provide the following information, where applicable:

- Accession codes, unique identifiers, or web links for publicly available datasets
- A description of any restrictions on data availability
- For clinical datasets or third party data, please ensure that the statement adheres to our policy

All data supporting the findings of this study are available within the main text and its Supplementary Information files. Raw data that supports the findings are available in the Source File or from the corresponding author upon reasonable request.

Human research participants

Policy information about studies involving human research participants and Sex and Gender in Research.

Reporting on sex and gender	*Use the terms sex (biological attribute) and gender (shaped by social and cultural circumstances) carefully in order to avoid confusing both terms. Indicate if findings apply to only one sex or gender; describe whether sex and gender were considered in study design whether sex and/or gender was determined based on self-reporting or assigned and methods used. Provide in the source data disaggregated sex and gender data where this information has been collected, and consent has been obtained for sharing of individual-level data; provide overall numbers in this Reporting Summary. Please state if this information has not been collected. Report sex- and gender-based analyses where performed, justify reasons for lack of sex- and gender-based analysis.*
Population characteristics	*Describe the covariate-relevant population characteristics of the human research participants (e.g. age, genotypic information, past and current diagnosis and treatment categories). If you filled out the behavioural & social sciences study design questions and have nothing to add here, write "See above."*
Recruitment	*Describe how participants were recruited. Outline any potential self-selection bias or other biases that may be present and how these are likely to impact results.*
Ethics oversight	*Identify the organization(s) that approved the study protocol.*

Note that full information on the approval of the study protocol must also be provided in the manuscript.

Field-specific reporting

Please select the one below that is the best fit for your research. If you are not sure, read the appropriate sections before making your selection.

☒ Life sciences ☐ Behavioural & social sciences ☐ Ecological, evolutionary & environmental sciences

For a reference copy of the document with all sections, see nature.com/documents/nr-reporting-summary-flat.pdf

Life sciences study design

All studies must disclose on these points even when the disclosure is negative.

Sample size	No statistical methods were used to predetermine sample size. Sample sizes were chosen to produce biologically meaningful data for comparison purposes, based on previous experiments of our laboratory and others using the physiological and biochemical methods described.
Data exclusions	No data were excluded from the analyses.
Replication	All experiments were conducted with at least two independent biological replicates and gave similar results.
Randomization	Sample randomization was not relevant for physiological and biochemical analyses in this study.
Blinding	Blinding was not performed. Group allocation according to genotype was done before data collection. Blinding was not possible because the same investigator processed the samples and analyzed the data.

Reporting for specific materials, systems and methods

We require information from authors about some types of materials, experimental systems and methods used in many studies. Here, indicate whether each material, system or method listed is relevant to your study. If you are not sure if a list item applies to your research, read the appropriate section before selecting a response.

Materials & experimental systems

n/a	Involved in the study	
☐	☒	Antibodies
☒	☐	Eukaryotic cell lines
☒	☐	Palaeontology and archaeology
☒	☐	Animals and other organisms
☒	☐	Clinical data
☒	☐	Dual use research of concern

Methods

n/a	Involved in the study	
☒	☐	ChIP-seq
☒	☐	Flow cytometry
☒	☐	MRI-based neuroimaging

Antibodies

Antibodies used

The anti-AHA2 polyclonal antibody (1:3000 dilution), anti-phosphorylated AHA2 polyclonal antibody (1:3000 dilution), anti-phot1 polyclonal antibody (1:3000 dilution), anti-GST antibody (1:3000 dilution) and anti-14-3-3 antibody (1:3000 dilution) had been produced by our research group. The anti-rabbit IgG antibody (goat) conjugated with HRP(1:3000 dilution, #170-6515) was purchased from Bio-Rad Laboratories, Inc.

Validation

The antibody profiles and validations are available in the following references.
Anti-AHA2and Anti-phosphorylated AHA2:
Hayashi, Y. et al. Biochemical Characterization of In Vitro Phosphorylation and Dephosphorylation of the Plasma Membrane H+-ATPase. Plant Cell Physiol. 51, 1186–1196 (2010).
Anti-phot1:
Emi, T., Kinoshita, T., Sakamoto, K., Mineyuki, Y. & Shimazaki, K. Isolation of a Protein Interacting with Vfphot1a in Guard Cells of Vicia faba. Plant Physiology 138, 1615–1626 (2005).
Anti-GST:
Hayashi, M., Inoue, S., Ueno, Y. & Kinoshita, T. A Raf-like protein kinase BHP mediates blue light-dependent stomatal opening. Sci. Rep. 7, 45586 (2017).
Kinoshita, T. & Shimazaki, K. Blue light activates the plasma membrane H+-ATPase by phosphorylation of the C-terminus in stomatal guard cells. EMBO J. 18, 5548–5558 (1999).
Anti-14-3-3:
Kinoshita, T. & Shimazaki, K. Blue light activates the plasma membrane H+-ATPase by phosphorylation of the C-terminus in stomatal guard cells. EMBO J. 18, 5548–5558 (1999).
